# Pitavastatin Calcium Confers Fungicidal Properties to Fluconazole by Inhibiting Ubiquinone Biosynthesis and Generating Reactive Oxygen Species

**DOI:** 10.3390/antiox13060667

**Published:** 2024-05-29

**Authors:** Wanqian Li, Yanru Feng, Zhe Feng, Li Wang, Malcolm Whiteway, Hui Lu, Yuanying Jiang

**Affiliations:** 1 Department of Pharmacy, Shanghai Tenth People’s Hospital, School of Medicine, Tongji University, Shanghai 200072, China; 2 Department of Biology, Concordia University, Montreal, QC H4B 1R6, Canada

**Keywords:** pitavastatin calcium, fluconazole tolerance, ubiquinone biosynthesis, reactive oxygen species, *Candida albicans*

## Abstract

Fluconazole (FLC) is extensively employed for the prophylaxis and treatment of invasive fungal infections (IFIs). However, the fungistatic nature of FLC renders pathogenic fungi capable of developing tolerance towards it. Consequently, converting FLC into a fungicidal agent using adjuvants assumes significance to circumvent FLC resistance and the perpetuation of fungal infections. This drug repurposing study has successfully identified pitavastatin calcium (PIT) as a promising adjuvant for enhancing the fungicidal activity of FLC from a comprehensive library of 2372 FDA-approved drugs. PIT could render FLC fungicidal even at concentrations as low as 1 μM. The median lethal dose (LD_50_) of PIT was determined to be 103.6 mg/kg. We have discovered that PIT achieves its synergistic effect by inhibiting the activity of 3-hydroxy-3-methylglutaryl-coenzyme A (HMG-CoA) reductase, thereby impeding ubiquinone biosynthesis, inducing reactive oxygen species (ROS) generation, triggering apoptosis, and disrupting Golgi function. We employed a *Candida albicans* strain that demonstrated a notable tolerance to FLC to infect mice and found that PIT effectively augmented the antifungal efficacy of FLC against IFIs. This study is an illustrative example of how FDA-approved drugs can effectively eliminate fungal tolerance to FLC.

## 1. Introduction

Invasive fungal infections (IFIs) present a significant health hazard to patients receiving hospital care, resulting in heightened rates of illness and death among individuals undergoing intricate surgical interventions or experiencing compromised immune function [1]. This issue is further compounded by increased drug-resistant fungal infections [2,3,4]. While novel approaches may offer potential solutions for treating IFIs [5,6], azoles, functioning as inhibitors of lanosterol 14-α demethylase, continue to hold paramount importance as clinically approved antifungal medications for managing these infections [7]. Fluconazole (FLC) is a prominent antifungal azole that is extensively utilized in clinical practice owing to its wide-ranging antifungal activity, remarkable safety record, and diverse modes of administration [8]. Nevertheless, the fungistatic nature of FLC renders pathogenic fungi capable of developing tolerance, characterized by FLC-susceptible fungal strains surviving at FLC concentrations higher than the minimum inhibitory concentration (MIC) [9]. It is imperative to render FLC fungicidal to effectively manage IFIs, as an increase in FLC tolerance leads to FLC resistance [10] and persistent candidemia [11,12].

One potential strategy for mitigating tolerance and resistance involves the utilization of combinations of FLC with an adjuvant, whereby the adjuvant exhibits a substantial enhancement in the effectiveness of FLC and may even render FLC fungicidal [8]. Several adjuvants, including cyclosporine A (CsA), a calcineurin inhibitor, geldanamycin (GdA), an Hsp90 inhibitor, rapamycin, a target of rapamycin (TOR) inhibitor, and brefeldin A, a guanine nucleotide exchange factor inhibitor, have exhibited the capacity to induce fungicidal effects on FLC in laboratory studies [11,13,14,15]. Nevertheless, their clinical utility is restricted due to their considerable toxicity or limited bioavailability. Despite the pressing need for the creation of safe and efficient novel pharmaceuticals to combat FLC tolerance in pathogenic fungi, the endeavor of developing an entirely new drug is a protracted and costly undertaking, necessitating a rigorous approval process by the Food and Drug Administration (FDA) [16]. Consequently, repurposing FDA-approved drugs presents an appealing alternative for developing FLC synergistic lethal adjuvants, as it circumvents the expenses associated with preclinical testing and leverages the established safety profiles and pharmacological attributes of these drugs. To investigate this possibility, we screened in vitro a collection of 2372 drugs approved by the FDA to find compounds capable of rendering FLC fungicidal.

Previously, the drug repositioning approach has successfully identified FLC adjuvants [17,18,19,20,21]. These studies commonly employed the fractional inhibitory concentration index (FICI) to assess the synergy between FLC and existing drugs, with a FICI value of ≤0.5 indicating synergy [22]. This method identified candidate compounds that effectively reduced MIC values of FLC in tested strains. However, the primary concern in treating IFIs using FLC is the occurrence of acquired drug resistance [10,11], as opposed to the insufficient blood concentration of FLC for effective antifungal activity. Therefore, in our high-throughput in vitro screening, the criterion for selecting FDA-approved drugs as FLC adjuvants was that the candidate drugs could make FLC fungicidal rather than reducing the MIC value of FLC.

In academic discourse, it has been widely believed that the effectiveness of FLC in treating IFIs is dependent on the level of FLC resistance exhibited by the pathogenic fungi, as indicated by the MIC value of FLC. Conversely, the tolerance level of these fungi towards FLC has been considered less influential [23]. In support, several studies found no positive relationship between FLC tolerance and mortality caused by IFIs after FLC treatment [24,25]. However, recent studies have suggested high FLC tolerance levels contribute to persistent candidemia [11,12]. Therefore, whether a high FLC tolerance level can undermine the antifungal effect of FLC and whether an FLC synergetic lethal adjuvant can enhance the efficacy of FLC against IFIs remains to be conclusively established.

Here, of 2372 existing FDA-approved drugs, we identified pitavastatin calcium (PIT) as the most promising adjuvant for FLC synergistic lethality through a high-throughput screen by broth microdilution, dose–matrix titration, disk diffusion, and acute toxicity assays. The synergistic fungicidal effect of PIT and FLC is contingent upon the inhibitory impact of PIT on 3-hydroxy-3-methylglutaryl-coenzyme A (HMG-CoA) reductase, thereby impeding the biosynthesis of ubiquinone, subsequently prompting the production of reactive oxygen species (ROS), initiating apoptosis, and perturbing Golgi function. PIT can potentially improve the effectiveness of FLC in treating invasive infections in mice caused by a strain that exhibits high tolerance to FLC. The findings of our study indicate that PIT could be repurposed as an adjunctive treatment to render FLC fungicidal, thereby augmenting its efficacy in managing IFIs caused by highly FLC-tolerant strains, such as *C. albicans*.

## 2. Materials and Methods

### 2.1. Strains, Primers, Agents, and Culture Conditions

Tables S1 and S2 present a comprehensive overview of the strains and primers utilized in this study. The *Candida* strains were cultivated under standard conditions in YPD media at a temperature of 30 °C, which consisted of yeast extract (1%), glucose (2%), and peptone (2%). A synthetic medium containing 2% dextrose and 6.7% yeast nitrogen base without amino acids was utilized for gene deletion. To prepare the media plates, a 2% agar was incorporated. A total of 2372 FDA-approved drugs were procured from MedChemExpress (MCE) in Shanghai, China. These drugs were individually dissolved in either water or dimethylsulfoxide (DMSO) to create stock solutions with a concentration of 10 mM. Subsequently, the 2372 drugs were stored separately in 96-well microplates at a temperature of −80 °C. Furthermore, drug stock solutions including FLC and CsA from Aladdin in Shanghai, China, PIT, domiphen bromide, ivermectin, triclosan, chlorhexidine digluconate, pimecrolimus, Dioscin, bleomycin sulfate, octenidine dihydrochloride, and vortioxetine from MCE in Shanghai, China, GdA from TargetMol, Boston, MA, USA, and (RS)-mevalonate lithium from Sigma in Shanghai, China were prepared using DMSO (Sangon Biotech, Shanghai, China) as the solvent; cerivastatin sodium and polymyxin B sulfate from TargetMol, USA were dissolved with distilled water; and ergosterol from Sangon Biotech in Shanghai, China was dissolved in Tween 80/ethanol (1:1).

### 2.2. High-Throughput Screening

The high-throughput screening was utilized to assess the impact of various compounds on *C. albicans*. *C. albicans* strain SC5314 was cultivated overnight at a temperature of 30 °C, following which a concentration of 4 μg/mL FLC was introduced to the cell suspension. This suspension was then serially diluted to 1 × 10^3^ cells/mL in a YPD medium. Subsequently, each well of the 96-well plates contained 198 μL of the cell suspension and 2 μL of one of the drugs from the library, resulting in an initial concentration of 100 μM. A total of 2 μL DMSO was added as the negative control. The 96-well plates were incubated for 48 h at a temperature of 30 °C. In order to ascertain the presence of synergistic lethal drugs, 5 μL of broth from each well that exhibited no observable growth during the screening assay was applied onto YPD recovery plates devoid of drugs. Synergistic fungicidal drugs were identified by observing the absence of colonies after a 48 h incubation period at 30 °C.

### 2.3. Antifungal Susceptibility Testing

The MIC assays for *C. albicans* were conducted following the broth microdilution protocol outlined by the Clinical and Laboratory Standards Institute (CLSI) protocols (M27, 4th edition). In brief, FDA-approved drugs were diluted in 96-well plates at two-fold the final concentration, with 4 μg/mL FLC. This dilution was combined with the cell suspension with a concentration of 1 × 10^3^ cells/mL. The plates were incubated at a temperature of 30 °C for 48 h without agitation. The MIC value was identified by the well with the lowest drug concentration in which the growth reduction, measured in terms of OD_600_ values, exceeded 50% compared to control cells without a compound.

A 5 μL broth from two neighboring wells that exhibited no observable growth in the microdilution assays was applied to drug-free YPD to evaluate the fungicidal properties of FLC and adjuvants. The establishment of synergistic fungicidal drugs was confirmed when no colonies were observed after 48 h at 30 °C.

The synergistic effects of drugs were conducted following the same procedure as the MIC assays [26]. Specifically, 50 μL of drug A was distributed through two-fold serial dilution across seven plate columns. Subsequently, 50 μL of drug B was distributed in a two-fold serial dilution manner along seven rows of the plate. Furthermore, 100 μL of *C. albicans* cultures, adjusted to a density of 1 × 10^3^ cells/mL, was dispensed into wells containing drugs and one control well devoid of any drugs. Before the 48 h OD_600_ readings, the plates were subjected to meticulous agitation to ensure the deposition of a representative 2 μL aliquot from each well onto newly prepared YPD recovery plates, thereby facilitating the evaluation of cellular recuperation following drug treatments. The recovery plates were subsequently incubated at a temperature of 30 °C for a duration ranging from 24 to 48 h, after which they were photographed. The experimental procedures were conducted in triplicate to ensure reliability and reproducibility.

### 2.4. Disk Diffusion Assays

Yeast cells were cultured overnight at a temperature of 30 °C, and the resulting cell suspension was diluted to a concentration of 1 × 10^7^ cells/mL in phosphate-buffered saline (PBS). At a concentration of 5 μM, the drugs were individually added to YPD plates for disk assays using a 25 μg FLC disk (6 mm, Liofilchem, Roseto degli Abruzzi, Italy). The plates were then incubated at 30 °C for 48 h. Subsequently, the disk was transferred to a drug-free YPD plate, which was incubated at 30 °C for 24 h and photographed [27].

### 2.5. Spotting Assay

The spotting assays were conducted following the previously described method [28]. In summary, *C. albicans* cultures were adjusted to a cell density of 1 × 10^7^ cells/mL using a hemacytometer. Subsequently, the cultures were serially diluted 1:10 and spotted onto a designated YPD solid medium containing specified concentrations of compounds. Following incubation at 30 °C for 48 h, photographs capturing the cell growth were taken, and the assays were replicated thrice.

### 2.6. Determination of MFC

The determination of MFC followed the previously described method [29]. In brief, a tested drug at a concentration two times higher than the final concentration was diluted in a two-fold serial dilution manner across ten columns of the plate, with each well containing 100 μL of the drug. Subsequently, 100 μL of *C. albicans* cultures (1 × 10^4^ cells/mL) were transferred into wells with and without any drugs as the control. Following incubation at 30 °C for 48 h, a 200 μL aliquot per well was removed and evenly spread on SDA plates, which were then incubated at 30 °C for 48 h. The MFC value was determined as the lowest drug concentration at which 99.9% of fungal colonies were eradicated compared to the control group.

### 2.7. Disruption, Ectopic Over-Expression, and Regulation of Target Genes

Target gene null mutants were constructed using fusion PCR to obtain homologous recombinant sequences, as previously described [30]. The *ADH1* promoter was utilized to upregulate target gene expression, while the DOX-inducible Tet-off promoter was employed to regulate target gene expression, following the methodology described earlier [31].

### 2.8. Quantitative Real-Time PCR (qRT-PCR) Analysis

To ensure the absence of genomic DNA contamination, the isolated RNA was subjected to DNase I treatment (TaKaRa, Dalian, China). First-strand cDNAs were synthesized using a reverse transcription PCR cDNA synthesis kit (Takara, China). Triplicate independent qRT-PCR analyses were performed using the Roche Lightcycler 96 Fluorescence Quantitative PCR Instrument and TB Green Premix Ex TaqTM II (Takara, China). The PCR protocol commenced with an initial step at 95 °C for 30 s, followed by 40 cycles comprising 95 °C for 5 s, 50 °C for 30 s, and 72 °C for 30 s. The *ACT1* gene served as the internal control [26].

### 2.9. Determination of Acute Toxicity of Compounds

The acute toxicity of compounds was assessed using the Up-and-Down procedure (UDP) as outlined in the OECD 425 guidelines, 2008. Following a previously described methodology, the intraperitoneal route was employed in adult mice [32]. In brief, the primary test involved a sequential administration of doses at intervals of at least 48 h, with one mouse being dosed at a time. Initially, a dose below LD_50_ was administered intraperitoneally to a mouse. If the mouse survived, the subsequent mouse would receive 1.3 times the initial dose. Conversely, if the mouse died, the subsequent mouse would receive a dose decreased by 1.3 times the initial dose. Each mouse was monitored for 48 h before determining the subsequent mouse’s dosage and administration. The experiment was terminated upon five “survive or die” reversals within a consecutive set of six animals tested. Subsequently, the LD_50_ and its corresponding confidence interval were computed based on the conditions observed in all mice after the experiment.

### 2.10. Measurement of ROS Generation

The *C. albicans* strain SN152 was cultured in a shaker at 30 °C for 16 h using a YPD medium. The culture was then diluted at a ratio of 1:100 and treated with 4 μg/mL FLC and 2 μM PIT for 4 h. The strains were stained with 5 mg/mL DCFH-DA or 100 nM MitoSOX Red (MCE, Shanghai, China) and incubated at 30 °C for 30 min. Subsequently, *C. albicans* cells were collected by centrifugation at 4000× *g* for 5 min and washed twice with sterile PBS. The intracellular and intra-mitochondrial ROS levels were measured using a BD FACSVerse flow cytometer (BD Biosciences, New York, NY, USA). The flow cytometer was used to detect the fluorescence intensities, with an excitation wavelength of 488 nm and an emission wavelength of 540 nm [29].

### 2.11. Caspase Assay6/4/2024

The strains that were cultured overnight were transferred at a dilution of 1:100 using a YPD medium. Following treatment with 4 μg/mL FLC and 2 μM PIT for 4 h, *C. albicans* cells were stained with (aspartyl)2-Rhodamine 110 (D_2_R) and incubated at a temperature of 30 °C for 45 min in the absence of light. Subsequently, the *C. albicans* cells were collected by centrifugation at 4000× *g* for 5 min and washed twice with sterile PBS. Before detection, each sample was adjusted to a concentration of 1 × 10^7^ cells/mL using sterile PBS. The activation of caspases was assessed using the Caspase Assay Kit (Flow cytometry) from BioVision, San Francisco, CA, USA. The fluorescence intensities were measured using flow cytometry with an excitation wavelength of 488 nm and an emission wavelength of 530 nm.

### 2.12. Cell Membrane Permeability Assay

The cell membrane permeability assay was conducted following a previously described method [29]. Briefly, *C. albicans* SN152 cells that had been cultured overnight were washed twice with sterilized PBS and adjusted to a concentration of 2 × 10^7^ cells/mL in 10 mL of PBS. The cell suspensions were then exposed to 4 μg/mL FLC alone, 2 μM PIT alone, and a combination of 2 μM PIT and 4 μg/mL FLC for 4 h at 30 °C. The group treated with 2 μM amphotericin B was used as the positive control, while the DMSO-treated group served as the negative control. Following treatment, the cell suspensions underwent centrifugation at 10,000 rpm for 10 min. The supernatant was subsequently collected, and the optical density was measured at wavelengths of 260 nm and 280 nm after filtration, utilizing a UV–vis spectrophotometer (Thermo Fisher Scientific, Waltham, MA, USA).

### 2.13. Murine Invasive C. albicans Infections

All studies utilized female C57BL/6 mice (6–8 weeks old) that were specific-pathogen-free and weighed between 17 and 19 g. All animals were purchased from Shanghai Slaccas Laboratory Animal Corporation (Shanghai, China), and all experimental procedures were performed according to the Animal Center of Tongji University guidelines. Before commencing drug therapy, the mice were inoculated with 200 μL of PBS containing 2 × 10^5^ *C. albicans* cells via the lateral tail vein. The mice were then administered intraperitoneal doses of either 1 or 4 mg/kg of FLC once a day for three consecutive days, along with intraperitoneal administration of PIT at doses of 1 or 3 mg/kg once a day for three consecutive days. The mice were weighed, and their weights were recorded every three days while their survival was monitored daily for 30 days. Two-tailed unpaired t-tests were employed to assess the weight changes. Kaplan–Meier analyses were employed to assess the survival probabilities, while log-rank testing was utilized to determine the statistical significance of the survival curves. In the case of fungal burden analyses, the mice were closely monitored twice daily for any indications of infection and were euthanized humanely upon reaching the predetermined endpoints. Subsequently, each mouse’s spleen, liver, and kidneys were harvested and homogenized in sterile PBS after sacrifice, followed by streaking onto SDA agar plates. Following a 48 h incubation period, the colonies were quantified, and colony-forming units (CFUs) were calculated. For histopathological analysis, kidney tissues from mice infected for 6 or 10 days were procured and fixed in buffered formalin. Subsequently, the tissues were embedded in paraffin, sectioned into 3–4 μm slices, and subjected to PAS staining using established protocols. All experimental procedures involving animals were approved by the Tongji University Animal Care Committee (No.: TJAA00322101).

## 3. Results

### 3.1. Screening FLC Synergetic Lethal Adjuvants from an FDA-Approved Drug Library

As part of a drug repositioning strategy, a high-throughput screen was conducted on an FDA-approved compound library of 2372 drugs to identify potential FLC-synergistic lethal adjuvants. The evaluation of these compounds involved assessing the synergistic lethal effect of 100 μM of each of the 2372 compounds, in combination with 4 μg/mL of FLC, on *C. albicans* SC5314 cultivated in a YPD medium within 96-well plates. After incubating at 30 °C for 48 h, *C. albicans* cells from wells exhibiting no visible cell growth were transferred onto a fresh YPD solid medium. It was observed that cells incubated with a combination of 222 compounds and FLC were unable to recover, whereas cells treated solely with FLC were able to recover (Table S3, Figure S1). We excluded 22 compounds that had been previously identified as having direct antifungal effects or fungicidal effects, including a polyene (Amphotericin B) [33], 15 azoles [7], 3 TOR inhibitors (Rapamycin, Everolimus, and Temsirolimus) [11,15], and 2 calcineurin inhibitors (Cyclosporin A and Tacrolimus) [34,35] (Table S3). Consequently, 200 compounds remained to be assessed for their potential synergistic lethal effects when combined with FLC.

**Figure 1:**
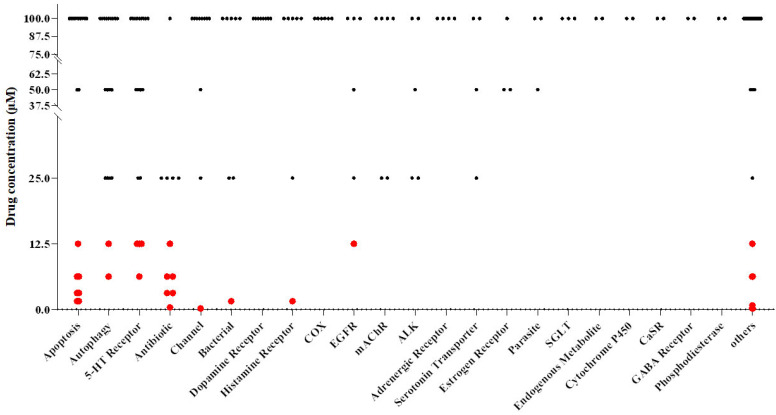
A screening process was conducted on the FDA-approved compound library, identifying 30 compounds that exhibit synergistic fungicidal properties against *C. albicans* when combined with FLC. The compounds exhibiting significant bioactivity at a synergistic lethal concentration lower than 12.5 μM are represented by red points.

Subsequently, we employed broth microdilution assays, incubated at a temperature of 30 °C for 48 h, to examine the synergistic lethality of coalescing 200 compounds within concentration ranges spanning from 100 μM to 0.1953 μM, with 4 μg/mL FLC. Subsequently, we transferred *C. albicans* cells from wells devoid of observable growth onto a YPD solid medium. Our findings revealed that 30 compounds could convert FLC from a fungistatic to a fungicidal state at concentrations below 12.5 μM (Figure 1, Table S3).

**Figure 2:**
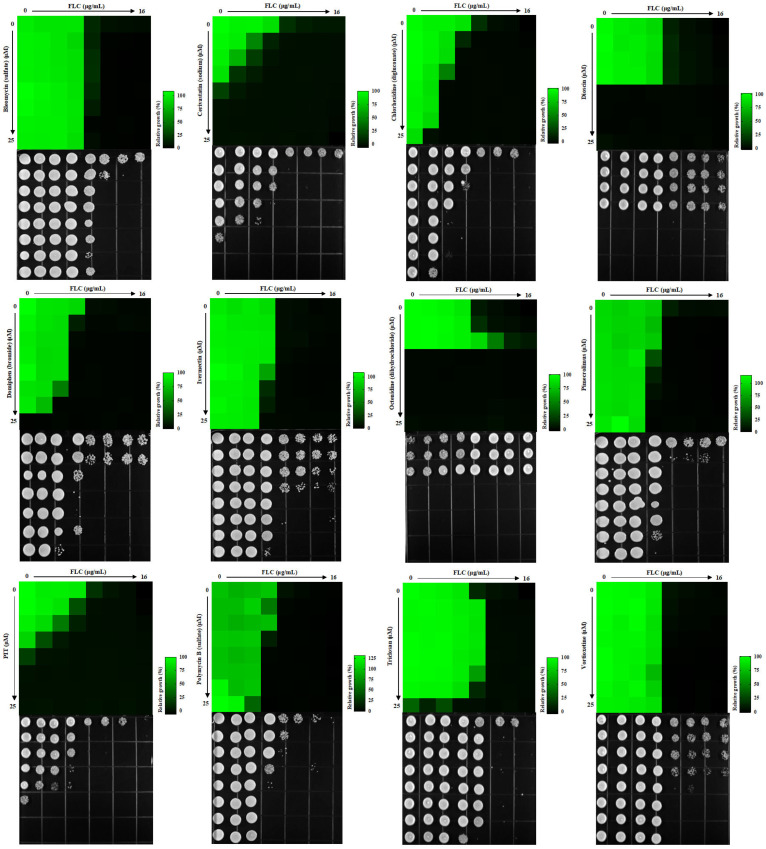
Twelve compounds eliminated the trailing growth of *C. albicans* exposed to FLC, each with a concentration below 3.125 μM. Dose–matrix titration assays were conducted using these 12 compounds and FLC in a YPD medium at 30 °C for 48 h.

The synergistic lethal activity of FLC and each of the 30 compounds was further confirmed through dose–matrix titration assays. FLC concentrations ranged from 16 μg/mL to 0.25 μg/mL, while the concentrations of the 30 compounds ranged from 25 μM to 0.7812 μM. We found that 12 compounds, at concentrations below 3.125 μM, could inhibit the trailing growth of *C. albicans* in the presence of FLC concentrations below 4 μg/mL (Figure 2). Subsequently, *C. albicans* cells were spotted onto a YPD solid medium. It was observed that cells treated with each of the 12 compounds, namely bleomycin sulfate, cerivastatin sodium, chlorhexidine digluconate, dioscin, domiphen bromide, ivermectin, octenidine dihydrochloride, pimecrolimus, PIT, polymyxin B sulfate, triclosan, and vortioxetine, in addition to FLC, displayed an inability to recover on YPD solid medium. Conversely, cells treated solely with FLC demonstrated the ability to recover (Figure 2). In conclusion, we have identified 12 FDA-approved drugs that, at concentrations ≤3.125 μM, can render FLC fungicidal at ≤4 μg/mL.

### 3.2. Eight Compounds Have Significant Synergistic Lethal Effects with FLC in Disk Diffusion Assays

The *C. albicans* FLC tolerance was evidenced by noticeable cell growth within the 25 μg FLC inhibition zone in the disk diffusion assays [11,36]. The FLC inhibition zones were clear when the YPD solid medium was supplemented with FLC synergistic lethal adjuvants such as GdA and CsA [34,37]. Therefore, we further assess the fungicidal potential of the 12 candidate FDA-approved drugs by visualizing their ability to convert FLC from fungistatic to fungicidal using FLC disk diffusion assays, with GdA and CsA as control compounds. For this analysis, we generated *C. albicans* mutants with elevated levels of FLC tolerance to compare the effectiveness of selected compounds and control drugs in clearing the tolerance of *C. albicans* to FLC. The *CMP1* gene (C1_00730C_A) encodes the catalytic subunit of calcineurin, which plays a critical role in *C. albicans* FLC tolerance. Removing the C-terminal autoinhibitory domain of Cmp1 can convert calcineurin into a constitutively activated complex [34,38,39]. Therefore, we deleted the C-terminal 110 residues of Cmp1 (*cmp1*-aid∆/*cmp1*-aid∆), creating a control strain with high FLC tolerance [34] (Figure S2A,B). Upc2 is a global ergosterol-synthesis-related gene regulator contributing to FLC tolerance [40,41]. To establish another control strain with high FLC tolerance, we utilized a P*_ADH1_*-*UPC2* strain [42], in which the *UPC2* gene (C1_08460C_A) was ectopically overexpressed under the regulation of the potent *ADH1* promoter [31] (Figure S2B).

**Figure 3:**
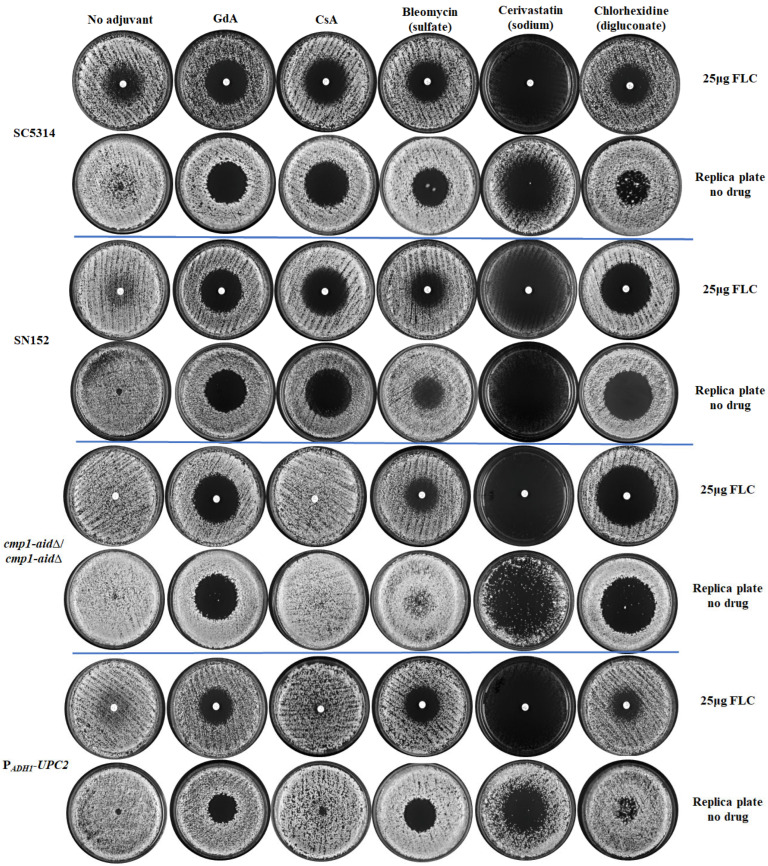

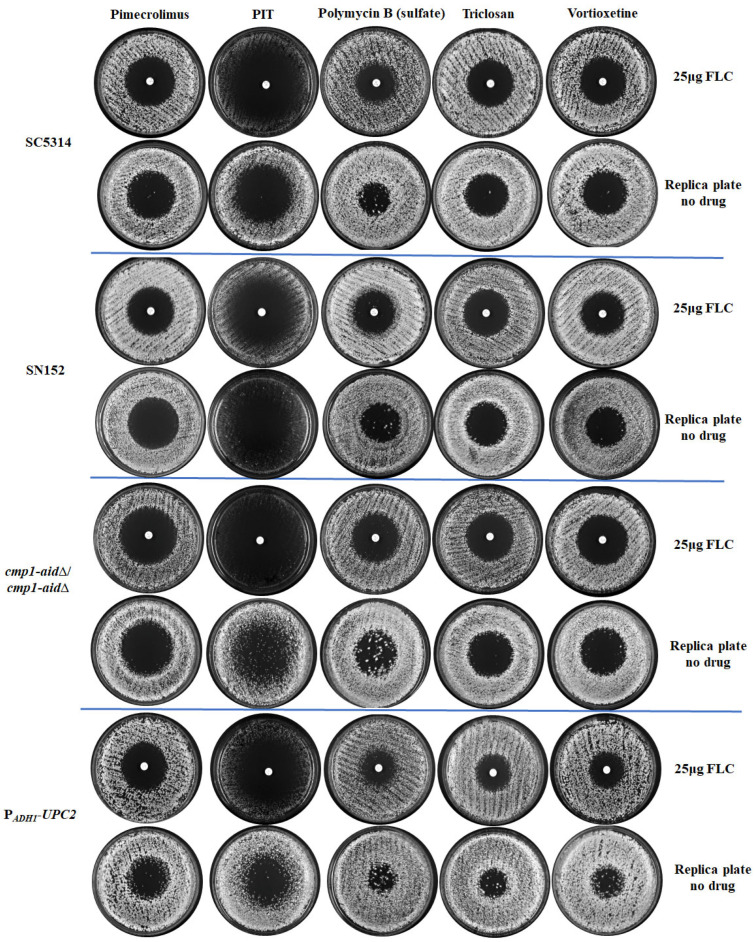
Eight compounds were identified from a pool of twelve candidate compounds to counteract the FLC tolerance exhibited by four strains subjected to testing. The FLC disk diffusion assays involved plating 1 × 10^6^ cells from a subset of strains with varying levels of FLC tolerance. These cells were grown on a YPD solid medium, both with and without adjuvant, at 30 °C for 48 h. Subsequently, the cells were replica plated onto YPD plates (devoid of FLC or adjuvants) and incubated at 30 °C for 24 h, following the removal of the FLC disk.

*C. albicans* SC5314, SN152, *cmp1*-aid∆/*cmp1*-aid∆, and P*_ADH1_*-*UPC2* strains with varying levels of FLC tolerance exhibited noticeable cell growth in the FLC inhibition zone. In comparison, GdA (5 μM) effectively eradicated the zones of FLC inhibition for all four strains (Figure 3). Conversely, CsA (5 μM) proved ineffective in eliminating FLC tolerance in both the *cmp1*-aid∆/*cmp1*-aid∆ and P*_ADH1_*-*UPC2* strains, exhibiting heightened FLC tolerance (Figure 3). Five compounds, cerivastatin sodium, pimecrolimus, PIT, triclosan, and vortioxetine, made FLC fungicidal against all four tested strains (Figure 3). However, chlorhexidine digluconate and polymyxin B sulfate were unsuccessful in reducing FLC tolerance in the P*_ADH1_*-*UPC2* strain, while bleomycin sulfate failed to clear the zone of FLC inhibition for the *cmp1*-aid∆/*cmp1*-aid∆ strain (Figure 3). Dioscin, domiphen bromide, ivermectin, and octenidine dihydrochloride were excluded based on their weaker synergistic lethal effects with FLC compared to GdA and CsA (Figure S3). Out of the initial 12 candidate compounds, a total of 8 compounds (bleomycin sulfate, cerivastatin sodium, chlorhexidine digluconate, pimecrolimus, PIT, polymyxin B, triclosan, and vortioxetine) were identified as either superior or equivalent to the control drugs in effectively eliminating FLC tolerance in the 4 strains under investigation.

### 3.3. PIT Has Low Toxicity

**Figure 4:**
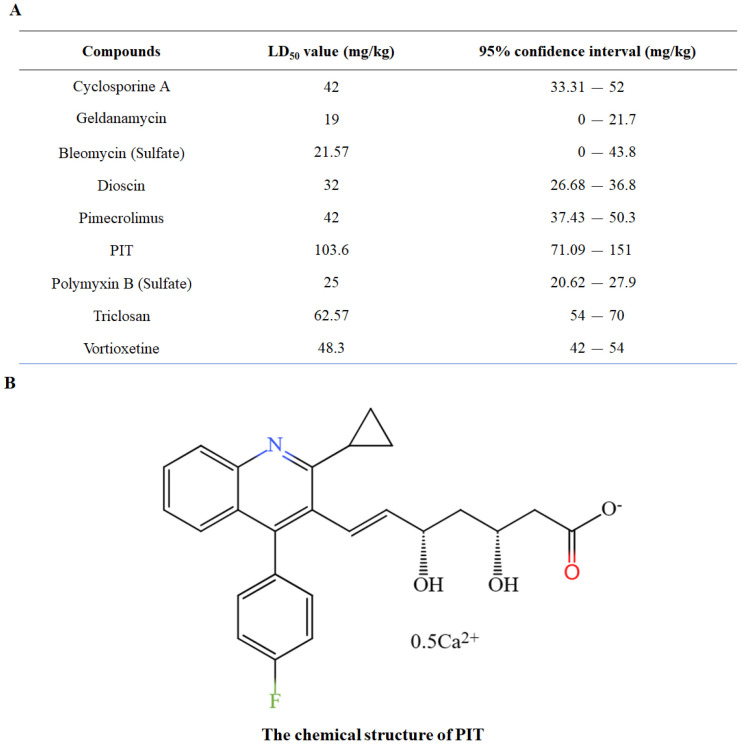
PIT has low toxicity. (**A**) The LD_50_ of nine compounds were determined using the Up-and-Down procedure (UDP) administered via the intraperitoneal route in mice. The certain adjuvants CsA and GdA were regarded as the control drugs. (**B**) The chemical structure of PIT, the candidate FLC adjuvant.

Although certain adjuvants, such as CsA and GdA, can make FLC fungicidal in laboratory studies, their clinical applications are limited due to their high toxicity [43,44]. Consequently, this study identifies adjuvants with reduced toxicity compared to CsA and GdA. The median lethal dose (LD_50_) of six compounds, which exhibited significant potential in clearing FLC tolerance based on previous screening, was determined using the Up-and-Down procedure (UDP) (OECD 425, 2008) administered via the intraperitoneal route in mice [32]. Cerivastatin sodium was excluded from the LD50 evaluation due to its documented association with severe rhabdomyolysis [45,46,47], while chlorhexidine digluconate was removed from consideration as it functions primarily as an external disinfectant. The LD_50_ values for GdA and CsA were determined to be 19 mg/kg and 42 mg/kg, respectively. In contrast, the LD_50_ values of bleomycin sulfate, polymyxin B Sulfate, pimecrolimus, and vortioxetine were either equivalent to or lower than those of the control drugs, indicating that these compounds possess comparable or heightened toxicity (Figure S4). The LD_50_ value of PIT (103.6 mg/kg) surpasses that of the control drugs (Figure 4A), signifying its superior toxicity profile compared to the candidate FLC adjuvants. Based on our initial compound collection, this observation suggests that PIT (Figure 4B) holds the greatest promise as a synergistic lethal agent for FLC.

### 3.4. PIT Confers Fungicidal Properties to Azoles

**Figure 5:**
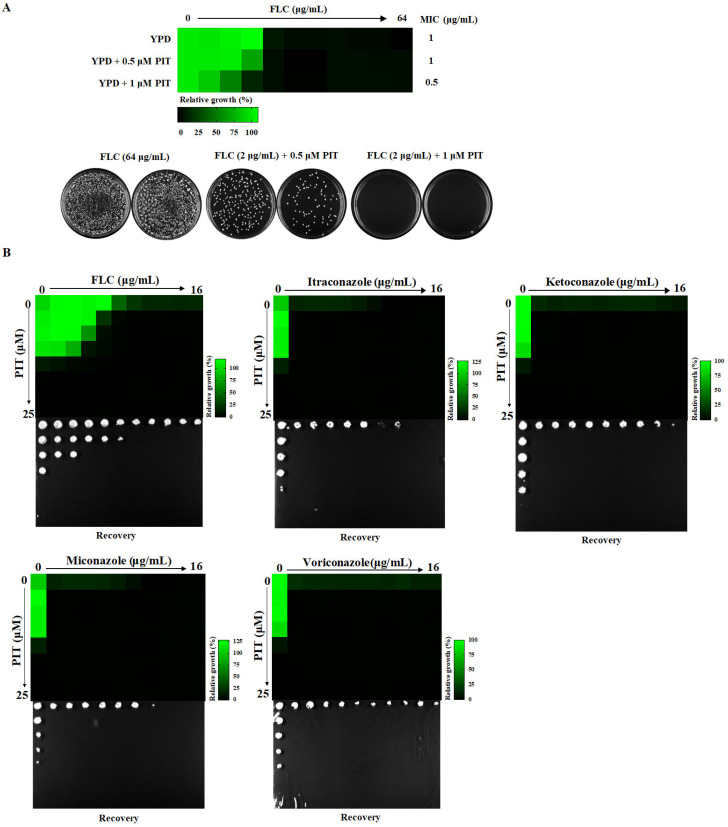

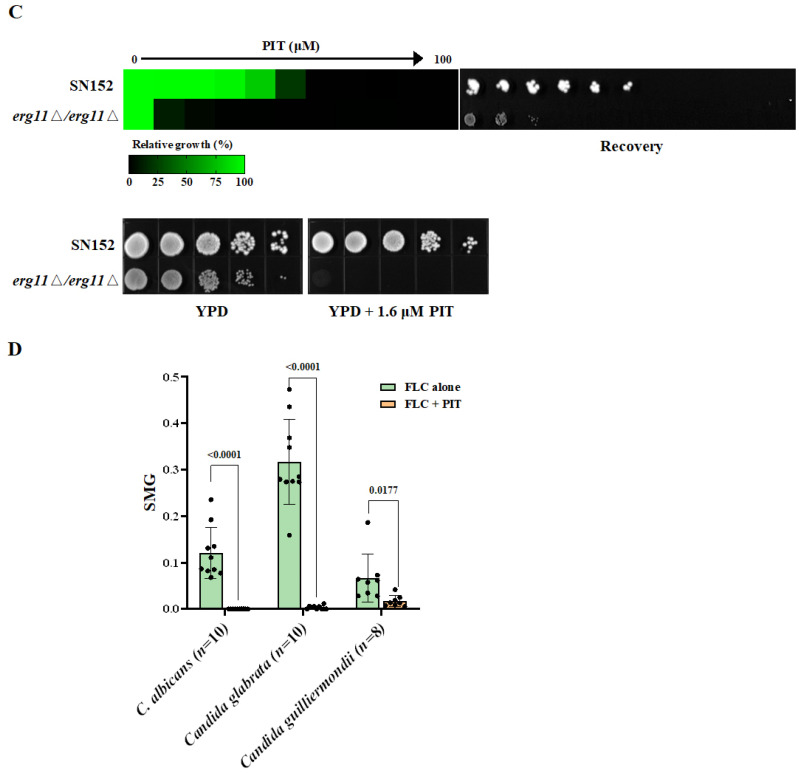
PIT confers fungicidal properties to azoles. (**A**) PIT makes FLC fungicidal. Two replicates are shown in the picture. (**B**) PIT confers fungicidal properties to voriconazole, ketoconazole, itraconazole, and miconazole. (**C**) MIC assays and spotting assays show that *erg11*∆/*erg11*∆ was significantly sensitive to PIT compared to the wild-type strain. (**D**) Supra-MIC growth (SMG) of FLC alone and in combination with PIT for *C. albicans*, *Candida glabrata*, and *Candida guilliermondii*.

The MIC value of FLC was determined to be 1 μg/mL, and the minimum fungicidal concentration (MFC) value of FLC was not detected in the absence of PIT, even at concentrations up to 64 μg/mL. However, in the presence of PIT (1 μM), the MIC value of FLC decreased to 0.5 μg/mL, and the MFC value decreased to 2 μg/mL. Consequently, the ratio of MFC to MIC in the presence of 1 μM PIT was 4 (Figure 5A), which aligns with the established definition of fungicidal activity (MFC/MIC ≤ 4) [48,49].

PIT combined with voriconazole, ketoconazole, itraconazole, and miconazole also showed synergistic lethal effects. Our findings revealed a significant enhancement in the antifungal activities of all these azoles when combined with PIT, rendering them fungicidal (Figure 5B). It is worth noting that azoles exert their antifungal effects by blocking the synthetic ergosterol pathway through direct inhibition of Erg11, a lanosterol 14α-demethylase encoded by the *ERG11* gene (C5_00660C_A) in *C. albicans*. Given the synergistic fungicidal activity of PIT and azoles, we speculated that the absence of the *ERG11* gene would lead to heightened sensitivity to PIT. To test this, we utilized an *erg11*∆/*erg11*∆ null mutant [42] and observed its MIC value of PIT lowered from 3.125 μg/mL in the control strain to 0.1953 μg/mL (Figure 5C), indicating that PIT has the potential to augment the antifungal efficacy of azoles. To further investigate the matter, we employed super-MIC growth (SMG) values to assess the impact of PIT on *Candida* clinical isolates that exhibited tolerance to FLC [11,42]. Our findings demonstrate that PIT significantly reduced the SMG values of FLC for a collection of clinical isolates. Specifically, the SMG value lowered from 0.1203 to 0 in *C. albicans* (*n* = 7) (*p* = 0.002); from 0.3169 to 0.0031 in *Candida glabrata* (*n* = 4) (*p* = 0.0442); and from 0.0667 to 0.0166 in *Candida guilliermondii* (*n* = 7) (*p* = 0.0362) (Figure 5D). These results indicate that PIT can eliminate the FLC tolerance observed in *Candida* clinical isolates.

### 3.5. PIT Makes FLC Fungicidal Depending on Targeting HMG-CoA Reductase

**Figure 6:**
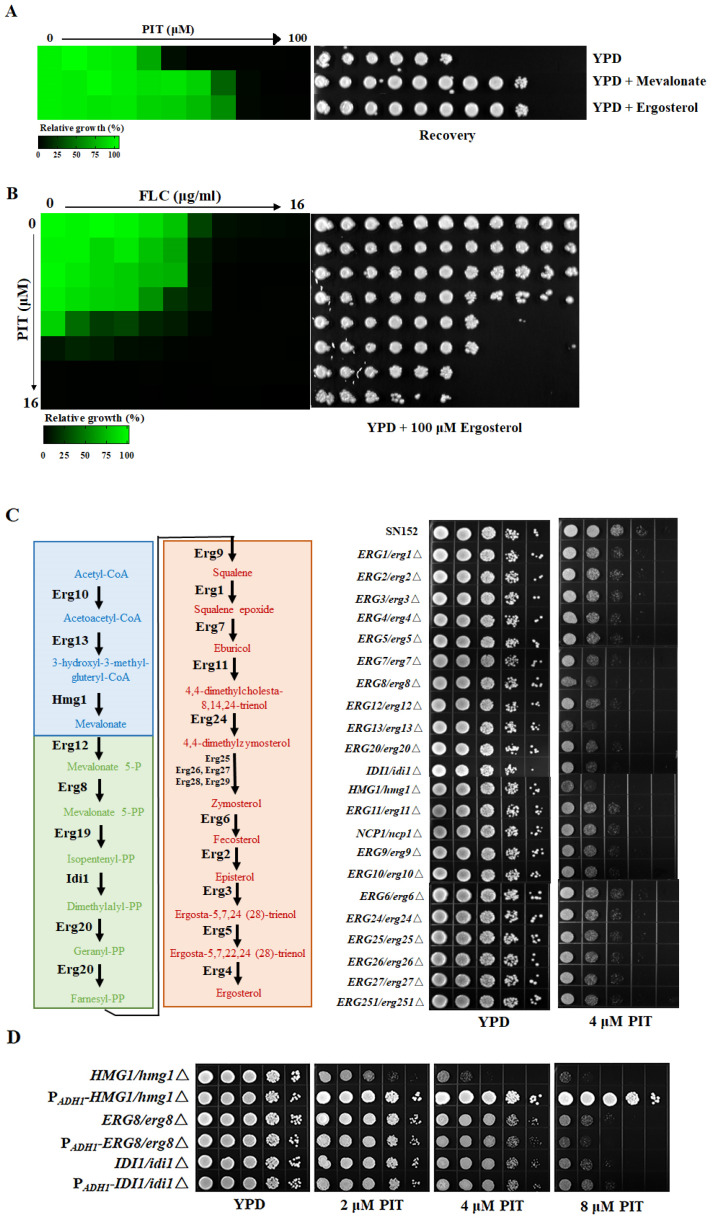
(**A**) MIC assays and spotting assays show that feeding 100 μM exogenous (RS)-mevalonate lithium or ergosterol could counteract the antifungal activity of PIT in *C. albicans* SN152. (**B**) Dose–matrix titration assays indicated that 100 μM ergosterol counteracted the antifungal activity of FLC and PIT. (**C**) (**Left**) The diagrammatic sketch of ergosterol biosynthesis pathway in *C. albicans*. The different colored boxes represent the three modules: the mevalonate pathway is the blue box; the green box is composed of farnesyl pyrophosphate biosynthesis; and the final orange box contains ergosterol biosynthesis. (**Right**) Compared to the SN152 strain and other heterozygous gene deletion mutants, the *HMG1*/*hmg1*Δ, *ERG8*/*erg8*Δ, and *IDI1*/*idi1*Δ mutants are more susceptible to 4 μM PIT. (**D**) Spotting assays were performed on the solid YPD medium containing different concentrations of PIT (0, 2, 4, and 8 μM). Only over-expressed *HMG1* gene could counteract the antifungal activity of PIT, rather than the *ERG8* and the *IDI1* genes.

Previous studies have indicated that statins, such as atorvastatin, lovastatin, and simvastatin, can suppress fungal ergosterol biosynthesis [50,51,52]. In our study, we observed that the antifungal efficacy of PIT was negated upon the introduction of 100 μM exogenous ergosterol (Figure 6A), and we also noted an upregulation in the expression of genes associated with ergosterol biosynthesis upon PIT treatment. We found that the mRNA expression levels of the *ERG1*, *ERG11*, and *ERG251* genes were upregulated 2-fold after 8 h of treatment with 2 μM PIT alone (Figure S5A–C). Simultaneously, when strains were conducted by 4 μg/mL FLC and 2 μM PIT for 8 h, the mRNA transcription level of the *ERG251* gene aggrandized about 6-fold, 3-fold of the *ERG1* gene and 2-fold of the *ERG11* gene compared with the control SN152 strain. However, compared with FLC treatment alone, only the mRNA transcription level of the *ERG251* gene aggrandized 4.57-fold (Figure S5D–F). These findings suggest that PIT exerts its antifungal properties by inhibiting ergosterol biosynthesis. Considering the dependence of PIT conferring FLC fungicidal on the reduction in ergosterol, we propose that the supplementation of ergosterol could mitigate the transition of FLC from fungistatic to fungicidal by PIT. Through dose–matrix titration assays, it was observed that 100 μM ergosterol, while not eliminating the synergistic lethal effect of FLC and PIT, significantly mitigated it (Figure 6B), supporting our conjecture.

To identify the potential target proteins of PIT involved in ergosterol biosynthesis, we constructed a series of heterozygous deletions of ergosterol biosynthesis-related gene mutants, including *ERG1*/*erg1*Δ, *ERG2*/*erg2*Δ, *ERG3*/*erg3*Δ, *ERG4*/*erg4*Δ, *ERG5*/*erg5*Δ, *ERG6*/*erg6*Δ, *ERG7*/*erg7*Δ, *ERG8*/*erg8*Δ, *ERG9*/*erg9*Δ, *ERG10*/*erg10*Δ, *ERG11*/*erg11*Δ, *ERG12*/*erg12*Δ, *ERG13*/*erg13*Δ, *ERG20*/*erg20*Δ, *ERG24*/*erg24*Δ, *ERG25*/*erg25*Δ, *ERG26*/*erg26*Δ, *ERG27*/*erg27*Δ, *ERG251*/*erg251*Δ, *HMG1*/*hmg1*Δ, *NCP1*/*ncp1*Δ, and *IDI1*/*idi1*Δ. We found that when compared to the wild-type strain and other heterozygous gene deletion mutants, the *HMG1*/*hmg1*Δ, *ERG8*/*erg8*Δ, and *IDI1*/*idi1*Δ mutants were more susceptible to 4 μM PIT (Figure 6C). We then over-expressed the *HMG1*, *ERG8*, and *IDI1* genes under the strong promoter PADH1 (Figure S6). We found that only the over-expressed *HMG1* gene, but not the *ERG8* or the *IDI1* genes, could counteract the antifungal activity of PIT (Figure 6D). Furthermore, the addition of 100 μM exogenous mevalonate, a byproduct of HMG-CoA catalyzed by HMG-CoA reductase, mitigated the antifungal effects of PIT (Figure 6A). These observations suggested that PIT targets HMG-CoA reductase encoded by the *HMG1* (C1_03780C_A) gene in *C. albicans* [50].

### 3.6. The Combination of PIT and FLC Elicits Reactive Oxygen Species (ROS) Generation by Inhibiting Ubiquinone Production

**Figure 7:**
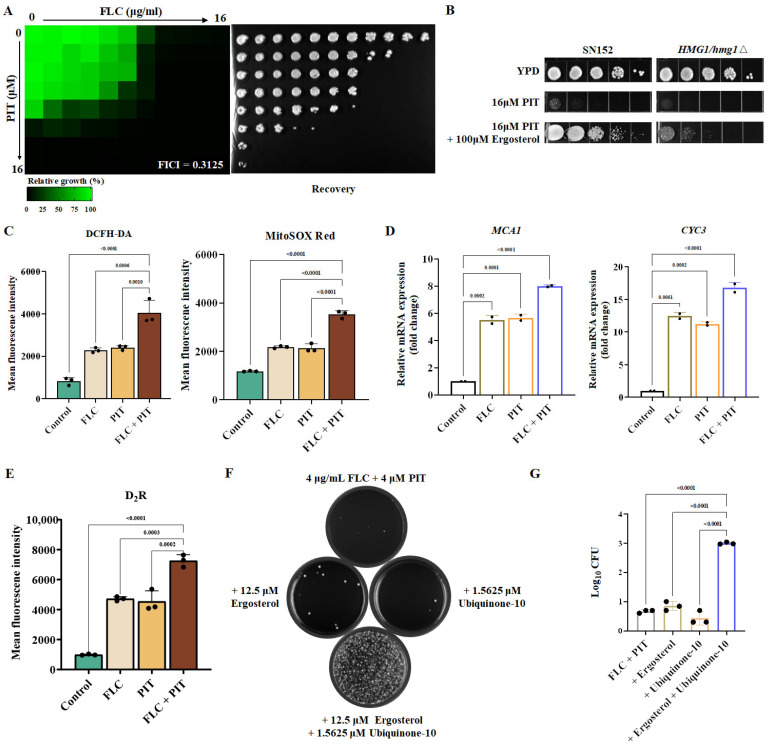
FLC, in combination with PIT, triggers ROS accumulation and induces apoptosis. (**A**) Dose–matrix titration assays hinted at the synergistic effect of FLC and PIT (FICI = 0.3125). (**B**) Spotting assays showed that the *HMG1*/*hmg1*∆ mutant was lethal under 16 μM PIT treatment and feeding 100 μM exogenous ergosterol could not fully regain the lethal activity. (**C**) Quantitative data of intracellular and mitochondrial ROS. After treatment with 4 μg/mL FLC and 2 μM PIT for 4 h, C. albicans cells were incubated with the fluorescent dye DCFH-DA or specific fluorescent dye MitoSOX Red at 30 °C for 30 min; then flow cytometry was employed. (**D**) The transcription levels of *MCA1* and *CYC3* in response to 4 μg/mL FLC and 2 μM PIT for 4 h were measured by qRT-PCR. (**E**) Determination of the caspase assay. After being treated with 4 μg/mL FLC and 2 μM PIT for 4 h, *C. albicans* cells were stained by (aspartyl)2-Rhodamine 110 (D2R) and analyzed by flow cytometry with excitation and emission wavelength at 488 and 530 nm, respectively. (**F**) Supplementation assays of ergosterol and ubiquinone. Dose–matrix titration assays indicated that 12.5 μM ergosterol and 1.5625 μM ubiquinone counteracted the antifungal activity of FLC and PIT. Then, the corresponding drug wells at that concentration were selected, and 100 μL per well was used to coat the YPD plate. The incubation time of the dose–matrix titration assays was 72 h, the incubation was continued for 48 h after coating the plates, and the incubation temperature was 30 °C in all cases. Three replications of this experiment were performed. (**G**) Quantification of supplementation assays of ergosterol and ubiquinone.

Does PIT exclusively act as an inhibitor of ergosterol synthesis to render FLC fungicidal? If PIT solely inhibits ergosterol synthesis and the antifungal mechanisms of PIT and FLC are identical, their interaction should be additive. However, our findings indicate that the two drugs display synergy, and FICI is 0.3125 (Figure 7A), implying that their antifungal mechanisms differ. Furthermore, ergosterol cannot fully restore the antifungal activity of PIT (Figure 7B). Consequently, we propose the hypothesis that PIT may possess alternative antifungal mechanisms in addition to its role as an inhibitor of ergosterol synthesis by targeting Hmg1 [53].

Many studies suggested that statins can cause mitochondrial dysfunction and stimulate the generation of ROS production [50,54]. Augmenting intracellular ROS levels in *C. albicans* offers a promising strategy for achieving fungicidal outcomes, like applying amphotericin B and miconazole, ultimately leading to cell demise [55]. Therefore, we postulate that the combination of PIT and FLC triggers ROS production, exerting a fungicidal effect. To verify our hypothesis, we initially used the fluorescent dye DCFH-DA to assess the levels of intracellular ROS. The findings demonstrated that intracellular ROS production had a 2-fold increase when the combination of PIT and FLC was applied (Figure 7C). Furthermore, we utilized MitoSOX Red, which selectively targets mitochondria, to evaluate the extent of mitochondrial ROS. The results indicated a 2-fold elevation in intra-mitochondrial ROS levels upon combining PIT and FLC (Figure 7C). The production of ROS is widely acknowledged to potentially initiate apoptosis [56]. Mca1 is a homolog of the *Saccharomyces cerevisiae* metacaspase Yca1, and its upregulation suggests mitochondria-mediated apoptosis. The upregulation of Cyc3 suggests the release of cytochrome c and the activation of metacaspase within the mitochondria [57]. The results of our study demonstrated a significant upregulation of approximately 7-fold in the mRNA expression of the *MCA1* gene compared to the control SN152 strain, and a 2.5-fold increase in comparison to FLC alone. Similarly, the mRNA expression of the *CYC3* gene showed a substantial upregulation of approximately 15.7-fold compared to the control and about 4.3-fold compared to FLC alone (Figure 7D). Notably, the caspase assay demonstrated a nearly 2-fold increase in caspase activity when FLC was combined with PIT, compared to FLC alone (Figure 7E), suggesting the initiation of mitochondria-mediated apoptosis. The combined treatment of FLC and PIT also resulted in the generation of ROS and subsequent induction of mitochondria-mediated apoptosis.

How does the PIT mechanism lead to ROS generation and the induction of mitochondrial dysfunction? PIT specifically targets HMG-CoA and inhibits mevalonate production, a precursor for both ubiquinone and ergosterol [58]. The reduction in ubiquinone levels subsequently results in the production of ROS and the impairment of mitochondrial function [59], potentially explaining the increased ROS production caused by PIT. Interestingly, it was observed that neither 12.5 μM ergosterol nor 1.5625 μM ubiquinone alone could compensate for the fungicidal effect of FLC in combination with PIT. However, when combined with ergosterol and ubiquinone, they could counteract the synergistic lethal effect of FLC and PIT (Figure 7F,G). Taken together, PIT targets HMG-CoA and suppresses ubiquinone synthesis, inducing ROS production and conferring fungicidal properties to FLC.

### 3.7. The Golgi Apparatus Exhibits Fragility When Subjected to a Combination of PIT and FLC

**Figure 8:**
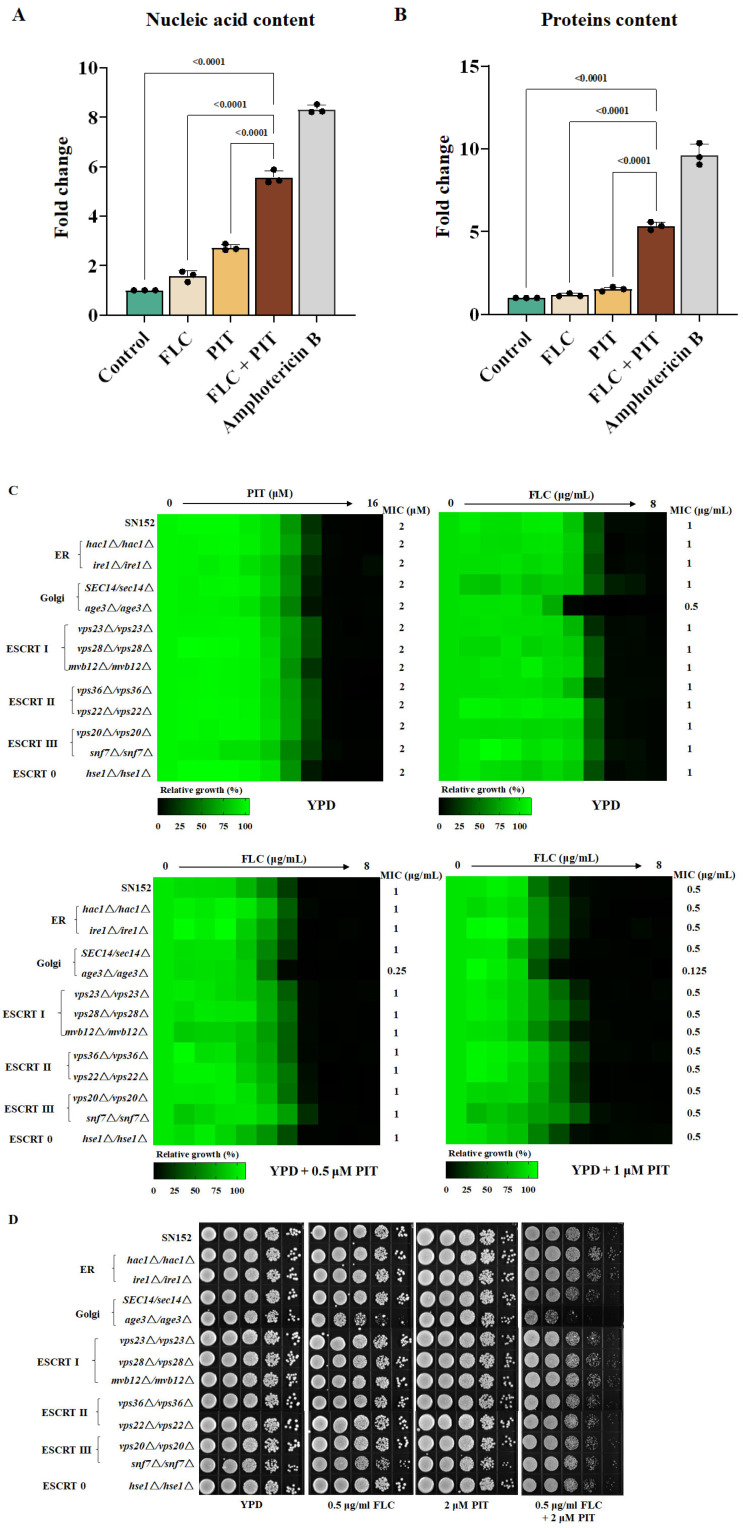
PIT made FLC fungicidal by disrupting membrane integrity. (**A**) The nucleic acid leakage in *C. albicans* SN152 after 4 μg/mL FLC, 2 μM PIT alone, and FLC and PIT combination for 4 h. Amphotericin B (2 μM) was regarded as the positive control. (**B**) The extracellular protein content in *C. albicans* SN152 after 4 μg/mL FLC, 2 μM PIT alone, and FLC and PIT combination for 4 h. Amphotericin B (2 μM) was regarded as the positive control. (**C**) MIC assays in YPD medium with PIT (0, 0.5, 1 μM) showed that the *age3*Δ/*age3*Δ mutant was more sensitive to FLC than other strains. (**D**) The *age3*Δ/*age3*Δ strain showed significant sensitivity to the combination of FLC and PIT using spotting assay analysis.

One aspect to consider is that ergosterol is a primary constituent of fungi plasma and organelle membranes. Consequently, it can be anticipated that inhibiting ergosterol biosynthesis through PIT and FLC treatment would disrupt the plasma and organelle membranes. Conversely, it is worth noting that ROS can initiate the oxidation process of proteins, lipids, and nucleic acids. This oxidation process can lead to compromised functionality of these essential biological macromolecules, ultimately causing damage to the membrane and triggering programmed cell death in the fungal pathogen [60,61]. The results of the cell membrane permeability assay indicate that the combined treatment of PIT and FLC significantly increased the extracellular release of nucleic acid and proteins compared to treatment with FLC alone. Specifically, the extracellular release of nucleic acid and proteins showed a 4.00 ± 0.17-fold (Figure 8A) and 4.18 ± 0.21-fold (Figure 8B) increase, respectively, suggesting disruption of the cell membrane. We wondered whether the combination of PIT and FLC causes indiscriminate damage to the membrane of fungal cells or specifically damages the membrane of certain organelles. Therefore, we constructed homozygous deletion mutants of the *HAC1* (*hac1*Δ/*hac1*Δ) (C1_06130C_A) and *IRE1* (*ire1*Δ/*ire1*Δ) (C1_07970C_A) genes, which play important roles in endoplasmic reticulum (ER) stress [62], and the *AGE3* (*age3*Δ/*age3*Δ) (C1_02260C_A) gene and a haploinsufficient mutant of *SEC14* (*SEC14*/*sec14*Δ)(C5_00480C_A), both of which are important for the Golgi transport network [13,63]. We also deleted *VPS23* (C1_10760W_A), *VPS28* (C2_08940C_A), and *MVB12* (CR_04790W_A) genes, which encode components of the endosomal sorting complexes required for transport (ESCRT) I subunit; *VPS36* (C2_04250W_A) and *VPS22* (C5_01620C_A) genes, which are important for ESCRT II; *VPS20* (C2_03310C_A) and *SNF7* (C1_00650C_A) genes, which encode two representative proteins of ESCRT III; and an *HSE1* (CR_01210C_A) gene, which is important for ESCRT 0 [64,65].

We found that the loss of these genes did not affect the antifungal effect of PIT, although removing the *AGE3* gene decreased the MIC value of FLC to 0.5 μg/mL, while the MIC value of FLC against the wild-type strain and other mutants was 1 μg/mL. Furthermore, 0.5 μM PIT reduced the FLC MIC to 0.25 μg/mL and 1 μM PIT reduced the FLC MIC to 0.125 μg/mL against the *age3*∆/*age3*∆ mutant (Figure 8C). However, compared to the wild-type *C. albicans* strain, PIT did not change the MIC value of FLC against other mutants (Figure 8C). Further spot assay showed that the *C. albicans* wild-type strain and the above mutants have similar susceptibility to FLC (0.5 μg/mL) and PIT (2 μM), and the *age3*∆/*age3*∆ mutant increased the susceptibility to the combination of FLC and PIT (Figure 8D). These observations suggest that PIT specifically impairs the Golgi membrane network.

### 3.8. PIT Enhances the Antifungal Efficacy of FLC against Invasive Candidiasis Caused by High FLC-Tolerant C. albicans Strains

The prevailing belief in the field is that the resistance of pathogenic fungi to FLC, rather than their tolerance to FLC, significantly impacts the effectiveness of FLC as an antifungal agent in vivo [23,24,25]. Thus, most studies focused on identifying FLC adjuvants aim to determine whether a compound can decrease the MIC value of FLC against pathogenic fungi, with little consideration given to its potential to render FLC fungicidal. However, an alternative perspective suggests that evaluating the antifungal efficacy of FLC and synthetic lethal adjuvants against IFIs is crucial when using strains that exhibit high tolerance to FLC [9,11]. The combination of PIT and FLC did not significantly enhance the antifungal activity against candidiasis caused by the low FLC-tolerance *C. albicans* SN152 strain in a mouse model (Figure S7).

**Figure 9:**
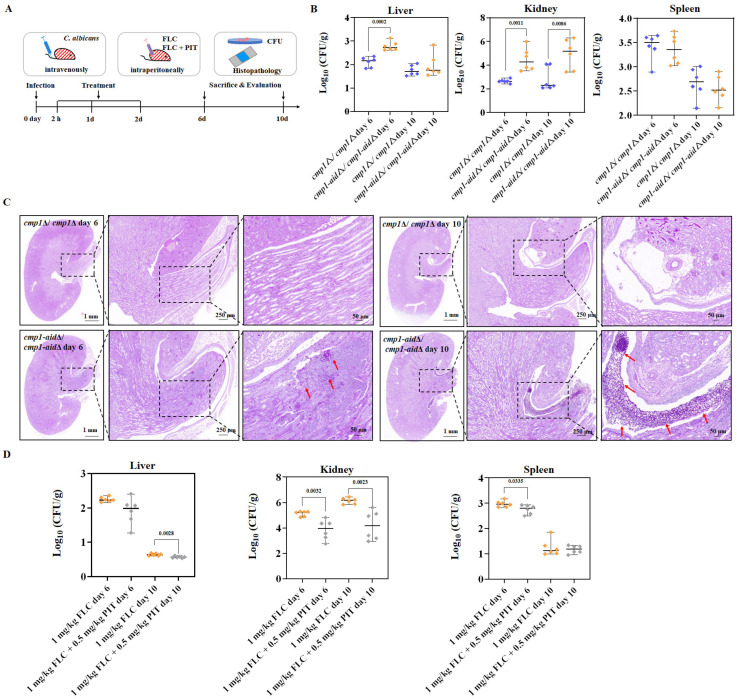
PIT enhances the antifungal activity of FLC against *C. albicans* in vivo. (**A**) Schematic overview of the timeline for the systemic candidiasis model. (**B**) CFUs analysis of infected liver, kidney, and spleen tissues after being infected for 6 or 10 days. C57BL/6 mice were intravenously infected with *cmp1*Δ/*cmp1*Δ (no FLC tolerance) and *cmp1*-aidΔ/*cmp1*-aidΔ (high FLC tolerance). FLC was administered at 1 mg/kg intraperitoneally once daily and lasted three days. CFUs were normalized to tissue weight (*n* = 6). *p* values were calculated using two-tailed unpaired *t*-tests. (**C**) Histopathology analysis of infected kidney tissues after 6 or 10 days of infection. Infected kidney tissues were stained with Periodic Acid-Schiff (PAS) and examined under ×1, ×3, and ×10 lenses (Scale bar = 1 mm, 250 μm, and 50 μm). Stained fungal cells are indicated with red arrows. Data were obtained from three independent experiments, and representative images are shown. (**D**) CFUs analysis of infected liver, kidney, and spleen tissues after 6 or 10 days of infection. C57BL/6 mice were intravenously infected with *cmp1*-aidΔ/*cmp1*-aidΔ. All values are CFUs recovered from tissue homogenates after 3 days of treatment. FLC was administered at 1 mg/kg and PIT at 0.5 mg/kg intraperitoneally once daily. CFUs were normalized to tissue weight (*n* = 6). *p* values were calculated using two-tailed unpaired *t*-tests.

Subsequently, the mutants *cmp1*Δ/*cmp1*Δ (no FLC tolerance) and *cmp1*-aidΔ/*cmp1*-aidΔ (high FLC tolerance) were utilized to infect C57BL/6 mice through tail vein injection (Figure 9A). Following a 2 h infection period, intraperitoneal administration of FLC (1 mg/kg) was initiated and continued for 3 days. After either 6 or 10 days following infection, six mice from each group were subjected to euthanasia to measure the burden of *C. albicans* in their kidneys, livers, and spleens. Subsequently, the kidney fungal burden was observed to be notably greater in the *cmp1*-aidΔ/*cmp1*-aidΔ mutant infected group compared to the *cmp1*Δ/*cmp1*Δ mutant infected group after 1 mg/kg FLC treatment (Figure 9B). In comparison to the *cmp1*Δ/*cmp1*Δ mutant infected group, the kidneys of the *cmp1*-aidΔ/*cmp1*-aidΔ mutant infected group exhibited an increased number of fungal infection lesions following FLC (1 mg/kg) treatment (Figure 9C). These findings indicate that the elevated FLC tolerance of *C. albicans* effectively compromises the in vivo antifungal efficacy of FLC. Subsequently, we infected mice with the *cmp1*-aidΔ/*cmp1*-aidΔ mutant and assessed the in vivo antifungal activity of the PIT and FLC combination. FLC (1 mg/kg) + PIT (0.5 mg/kg) significantly reduced the fungal burden in the kidney (*p* = 0.0032 after being infected for 6 days, *p* = 0.0023 after being infected for 10 days, *t*-test) in comparison to FLC (1 mg/kg)-treated groups after infections of 6 or 10 days (Figure 9D), suggesting PIT enhanced the antifungal activity of FLC against a highly tolerant strain of *C. albicans*.

**Figure 10:**
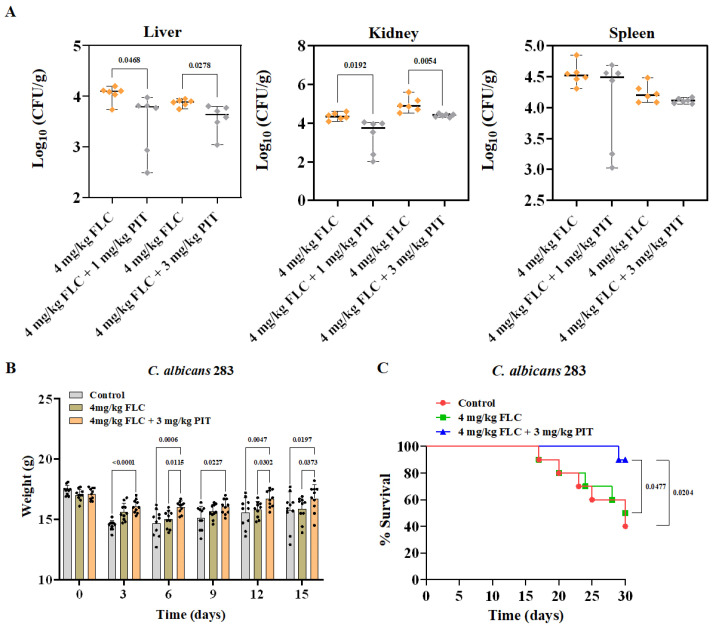
PIT strengthens the antifungal activity of FLC against clinically relevant *C. albicans* in vivo. (**A**) CFU analysis of infected liver, kidney, and spleen tissues after 6 days of infection. C57BL/6 mice were infected with 283 cells of *C. albicans* via tail vein injection. All values are CFUs recovered from tissue homogenates after 3 days of treatment. FLC was administered at 4 mg/kg and PIT at 1 or 3 mg/kg intraperitoneally once daily. CFUs were normalized to tissue weight (*n* = 6). *p* values were calculated using two-tailed unpaired *t*-tests. (**B**) The weight of C57BL/6 mice was monitored once every 3 days for 15 days. Two-tailed, unpaired *t*-tests and two-way ANOVAs were used. (**C**) C57BL/6 mice were monitored for the health and survival of treated (green and blue) and untreated (red) mice for 30 days. Significance was determined using the log-rank (Mantel–Cox) test.

To assess the potential enhancement of the antifungal activity of FLC against clinically relevant *C. albicans* in vivo, we utilized the mouse candidiasis model and a clinical *C. albicans* isolate with high FLC tolerance, specifically *C. albicans* isolate 283. C57BL/6 mice were infected with 283 cells of *C. albicans* through tail vein injection. Following a 2 h infection period, intraperitoneal administration of FLC (4 mg/kg) and FLC (4 mg/kg) + PIT (1 or 3 mg/kg) treatment was administered for 3 days. After 6 days of infection, six mice from each experimental group were euthanized, and their kidneys were examined to determine the burden of *C. albicans*. The results indicated a significant reduction in fungal burden after treatment with FLC + PIT (*p* = 0.0192 for 1 mg/kg PIT, *p* = 0.0054 for 3 mg/kg PIT, *t*-test) in comparison to the group treated solely with FLC (Figure 10A).

To assess the therapeutic potential of the PIT and FLC combination, we conducted a randomized division of mice into three groups: (1) a control group receiving no drug treatment, (2) a group treated with 4 mg/kg FLC, and (3) a group treated with 4 mg/kg FLC in combination with 3 mg/kg PIT. Each group consisted of ten mice. The protection of mice from *C. albicans* infection because of the FLC and PIT combination was evaluated by measuring the body weights of the mice. At the onset of infection (day 0), no significant differences were observed in the average body weight among the three groups, and within 15 days following infection, the control and FLC-treated groups exhibited greater weight loss than those receiving combined FLC and PIT treatment (Figure 10B). Specifically, the average weight losses on the 15th day of infection were 1.65 ± 1.21 g, 1.16 ± 0.63 g, and 0.39 ± 0.69 g for the control, FLC-treated, and FLC plus PIT-treated groups, respectively. Furthermore, our observations have revealed a mortality rate of 60% in the control group throughout the 30-day observation period. However, upon administration of 4 mg/kg FLC, the mortality rate of infected mice decreased to 50%. The administration of PIT (3 mg/kg) significantly augmented the antifungal efficacy of FLC (4 mg/kg) against *C. albicans* infection, as evidenced by the reduction in mortality rate to 10% in the FLC plus PIT-treated group (*p* value = 0.0204, compared to the control group; *p* value = 0.0477, compared to the FLC-treated group; assessed using the log-rank test) (Figure 10C). These in vivo experiments prove that PIT eliminates FLC tolerance and enhances its antifungal activity against *C. albicans* infection.

## 4. Discussion

**Figure 11:**
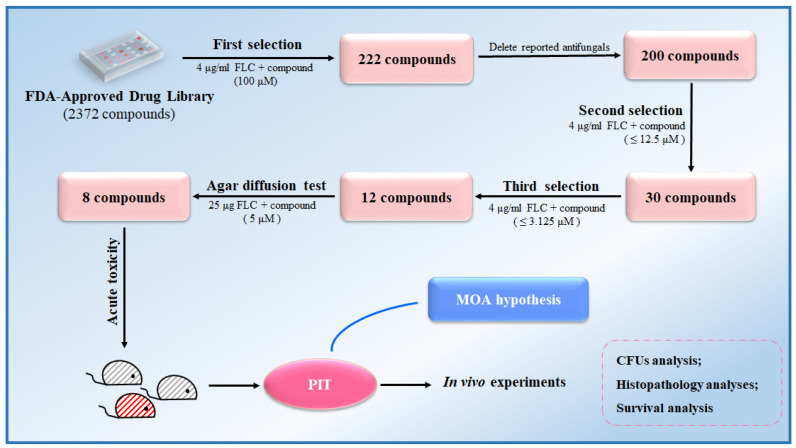
Identification of PIT as a promising FLC synergistic lethal adjuvant, thereby augmenting the efficacy of FLC in managing IFIs caused by highly FLC-tolerant strains.

As shown in Figure 11, following a drug repurposing strategy, we screened an FDA-approved compound library. We identified PIT as a promising FLC synergistic lethal adjuvant, which can make FLC fungicidal against *C. albicans* at a very low concentration and has low toxicity. Our study suggests that PIT plays its antifungal role by targeting Hmg1, inhibiting ergosterol synthesis, and generating ROS, primarily damaging the Golgi function, ultimately leading to apoptosis and cell demise. PIT enhanced the efficacy of FLC against invasive candidiasis in mice caused by highly FLC-tolerant *C. albicans* strains by making FLC fungicidal.

Drug repurposing was widely used to find new antifungal agents, especially identifying new antifungal applications for FDA-approved drugs [66]. The advantages of drug repurposing include a lower risk of failure, especially in safety and pharmacokinetic parameters, and a shorter time for evaluating the efficacy of an old drug [67]. Some FDA-approved drugs previously identified for FLC adjuvants were repurposed to treat fungal infections [17,68]. For example, ibuprofen, a non-steroidal anti-inflammatory drug, demonstrated synergy with FLC against *C. albicans* in vitro and in vivo [69,70,71,72]. However, most of these studies used FICI as the evaluation standard to determine FDA-approved drugs that reduced the MIC value of FLC and did not elevate the synergistic lethal effect of the combinations of FDA-approved drugs and FLC. The importance of rendering FLC fungicidal in treating IFIs arises from the increasing prevalence of acquired FLC resistance and the persistent nature of fungal infections attributed to the fungistatic properties of FLC [10,11,12]. Our present study provides a new high throughput way to identify FLC adjuvants; that is, the candidate adjuvants should make FLC have a fungicidal effect and find that PIT is a synergistic lethal FLC adjuvant.

It has been observed that statins are beneficial for FLC against *Candida* species. For example, fluvastatin acts synergistically with FLC against *Candida* species [73,74,75], including FLC-resistant strains of *C. albicans* and *C. tropicalis* [74,76]. In addition, simvastatin synergistically increased the antifungal effect of FLC against some clinical isolates of *Candida* species [77]. Likewise, lovastatin can act synergistically with FLC against *C. albicans* and *C. glabrata* [75,78]. Atorvastatin also increases the anti-*C. albicans* efficacy of FLC [73]. In the case of rosuvastatin and FLC, the combination is effective against *C. albicans* [73]. A recent study demonstrated that pitavastatin as a potent azole chemosensitizing agent remarkably boosted the effects of FLC against azole-resistant *Candida* species [20]. Lastly, pravastatin exhibits synergistic effectiveness with FLC against *C. albicans* only [79]. These findings suggested that statins are potential adjuvants that can enhance the efficacy of FLC in the anti-Candida species. However, these studies only incorporated one or two statins in conjunction with FLC, and a comprehensive evaluation of the optimal combinations of statins and FLC was not conducted. Here, we systematically evaluated the synergetic lethal effects of 12 statins (atorvastatin, atorvastatin hemicalcium, bestatin, bestatin hydrochloride, cerivastatin sodium, cilastatin, fluvastatin sodium, lovastatin, pentostatin, PIT, pravastatin, and rosuvastatin) and FLC against *C. albicans*. Still, only three statins (cerivastatin sodium, fluvastatin sodium, and PIT) enhanced the antifungal activity of FLC at concentrations ≤ 6.25 μM. In contrast, most of the tested statins had no synthetic lethal effects with FLC at a tested concentration ≥ 50 μM (Figure S8). These results suggested that PIT could more specifically target the Hmg1 of *C. albicans* than other statins.

The chemosensitizing activity of PIT in the FLC has been ascribed to its capacity to selectively target Hmg1, disrupt the biosynthesis of ergosterol and coenzyme Q, and induce the generation of ROS. Notably, the susceptibility of *C. albicans* to PIT was heightened by the heterozygous deletion of the *HMG1* gene, while the overexpression of the *HMG1* gene attenuated the antifungal efficacy of PIT. It is important to note that while this study did not directly measure intracellular ergosterol levels, the inhibition of ergosterol synthesis is supported by the fact that the antifungal activity of PIT was reversed by the administration of exogenous mevalonate. Previous studies suggested that lovastatin and pravastatin disrupted mitochondrial function due to media with glucose as a carbon source, which exhibited minor growth inhibition. The inhibition is significantly enhanced in media with ethanol as the carbon source [78,79]. We found that FLC and PIT promote ROS production and subsequent induction of mitochondria-mediated apoptosis. Furthermore, we found that loss of Age3 enhanced the antifungal efficacy of the combination of FLC and PIT, suggesting that PIT damaged the Golgi membrane network through inhibition of ergosterol biosynthesis.

Many compounds have been found to make FLC fungicidal, such as GdA, CsA, brefeldin A, and so on [13,28,35,37]. Unfortunately, there are few FLC synergistic lethal adjuvants for clinical use. One reason is that these adjuvants can have serious toxic effects [43,44,57]. More importantly, the synergistic effect of these compounds on FLC in vitro has yet to be verified in vivo [23,24]. Recent studies show that persistent candidemia is linked to a significant tolerance level towards FLC [11,12], suggesting that the antifungal activity of FLC synergistic lethal adjuvants should be evaluated by infecting mice with strains with a high FLC tolerance level. We infected mice using one pair of *C. albicans* strains with or without FLC tolerance (the *cmp1*-aidΔ/*cmp1*-aidΔ mutant vs. the *cmp1*Δ/*cmp1*Δ mutant) and evaluated the efficacy of FLC against IFIs. Indeed, we found that after a short period of FLC treatment, there was more fungal colonization in the kidneys of mice infected with the *cmp1*-aidΔ/*cmp1*-aidΔ mutant compared with those infected with the *cmp1*Δ/*cmp1*Δ mutant, suggesting that high FLC tolerance did weaken the antifungal activity of FLC against *C. albicans*. These results suggest that the combination of FLC and adjuvant has therapeutic advantages over FLC monotherapy only when it is used to treat IFIs caused by *C. albicans* strains with high FLC tolerance levels.

## Data Availability

The data are contained within the article and Supplementary Materials.

## Supplementary Materials

The following supporting information can be downloaded at: https://www.mdpi.com/article/10.3390/antiox13060667/s1.
