# Supplementary material for: Pitavastatin Calcium Confers Fungicidal Properties to Fluconazole by Inhibiting Ubiquinone Biosynthesis and Generating Reactive Oxygen Species
Source: Antioxidants (Basel). 2024 May 29;13(6):667. doi: 10.3390/antiox13060667 (PMC11200976; doi:10.3390/antiox13060667)
Supplement: Supplementary file 1 [file antioxidants-13-00667-s001.zip › Supplementary Figures Revised.pdf]

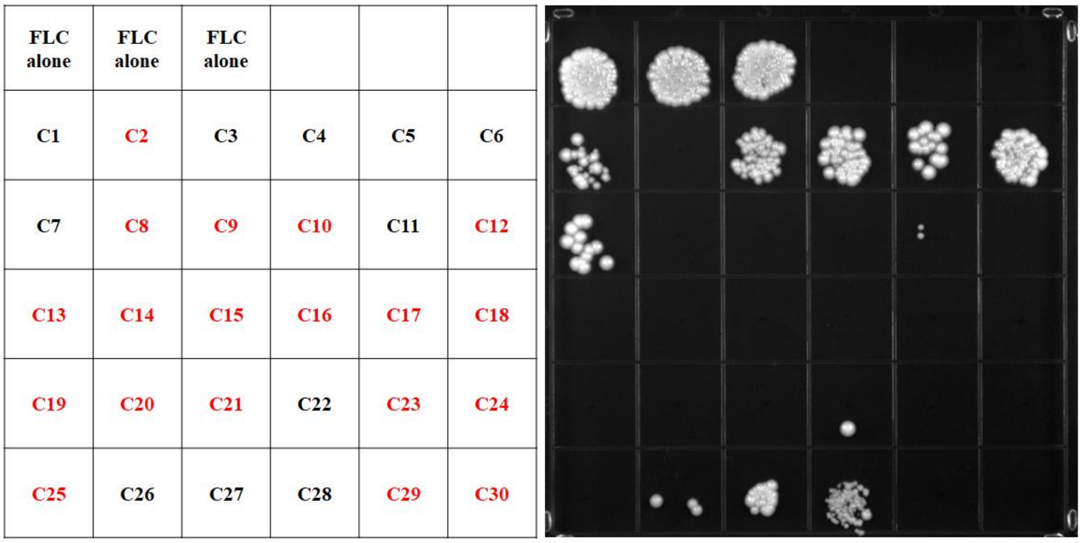

Figure S1. A screening criterion of a high-throughput screen to identify potential FLC-synergistic lethal adjuvants. Broth microdilution assays with 2372 compounds (100 μM) and 4 μg/mL FLC were performed in YPD medium in 96-well plates on *C. albicans* SC5314. After incubating for 48 h at 30 °C, 5 μL *C. albicans* cells from wells where no cell growth was visible onto YPD solid medium. Taking 30 of 2372 compounds as examples, cells treated with 19 compounds (red font) plus FLC did not recover, while cells treated simply with FLC did. Then, the 19 compounds will be candidates to be further evaluated.

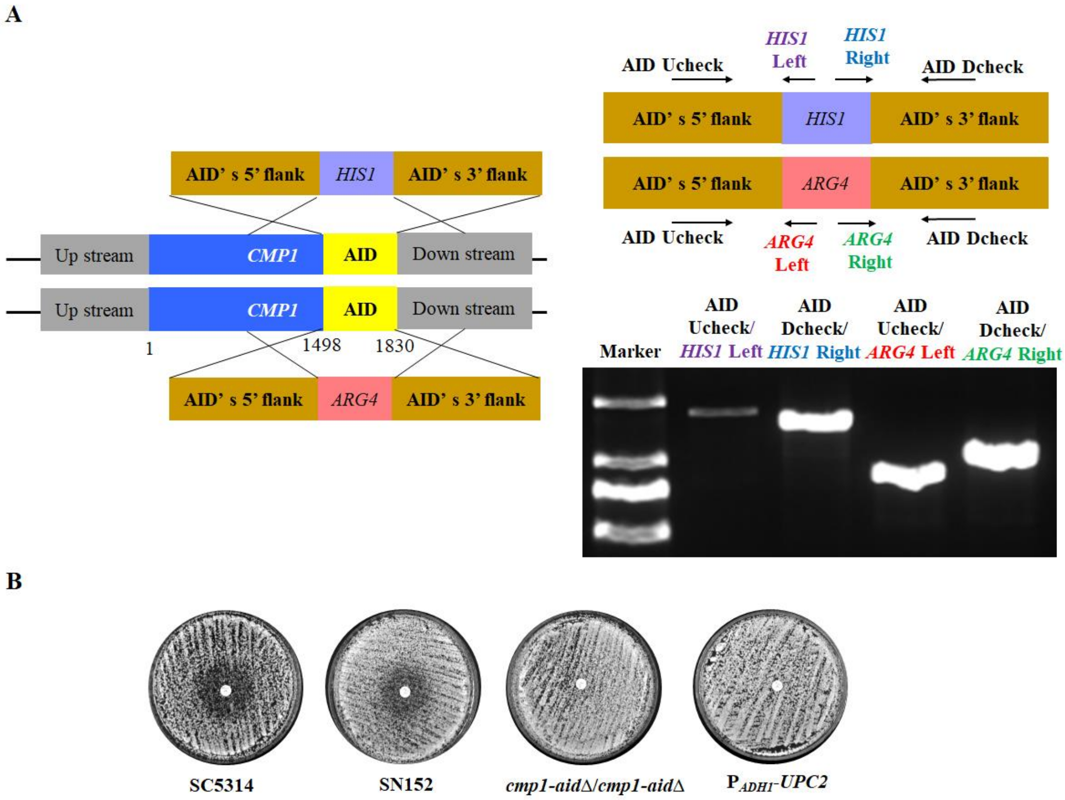

Figure S2. Construction of the homozygous deletion of *cmp1-aid*Δ/*cmp1-aid*Δ. **(A)** The schematic diagram of the *cmp1-aid*Δ/*cmp1-aid*Δ gene disruption and PCR verification results of the homozygous deletion of *cmp1*Δ/*cmp1*Δ and *cmp1-aid*Δ/*cmp1-*

*aid*Δ. **(B)** FLC disk diffusion assays of *cmp1-aid*Δ/*cmp1-aid*Δ and P_{ADH1}-*UPC2*.

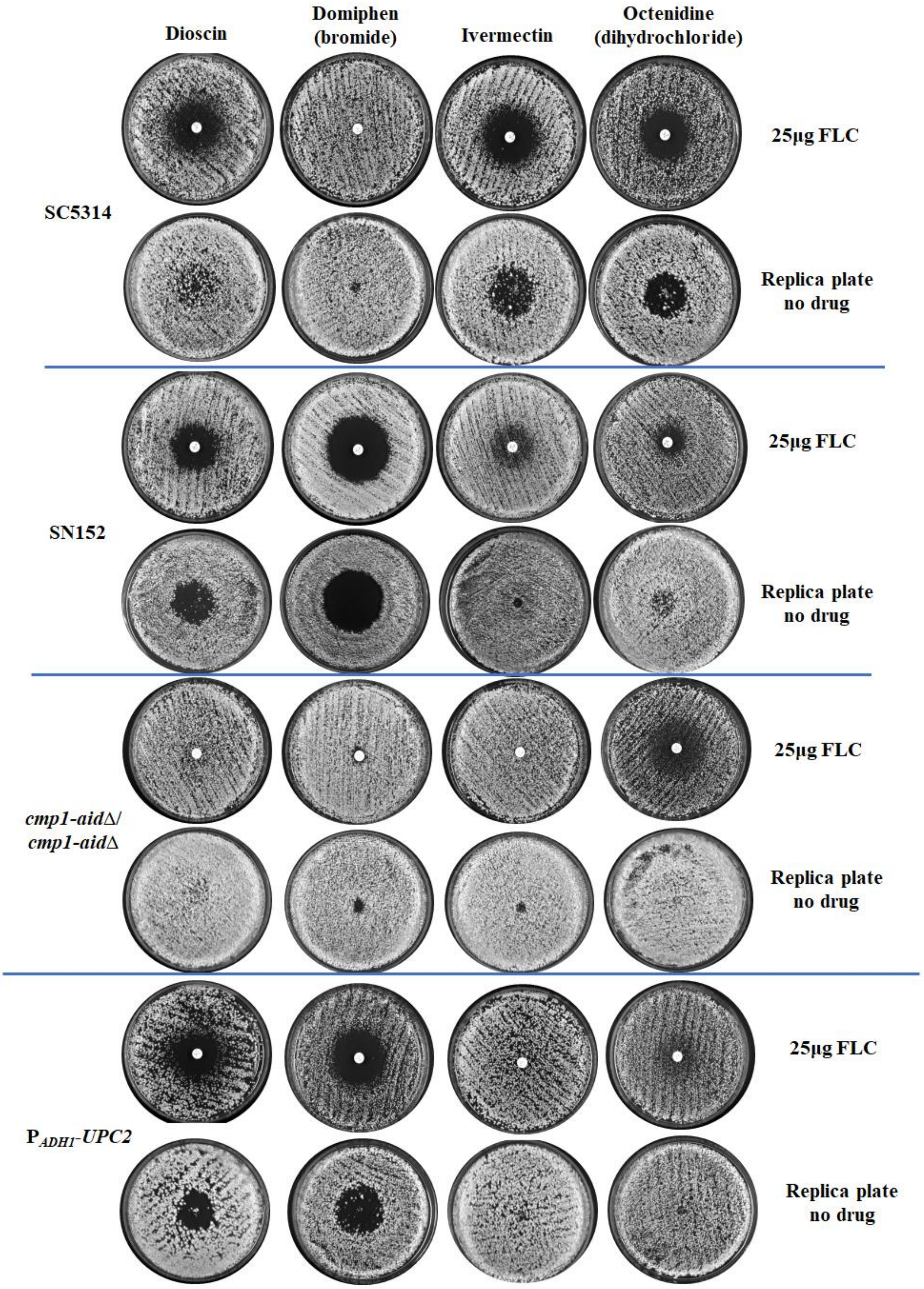

Figure S3. FLC disk diffusion assays of the remaining four compounds, including dioscin, domiphen bromide, ivermectin, and octenidine dihydrochloride.

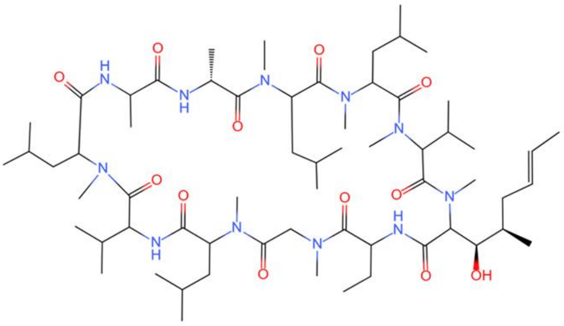

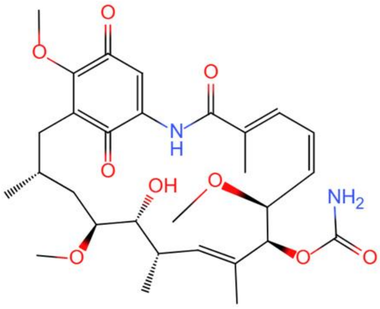

Cyclosporine A

Geldanamycin

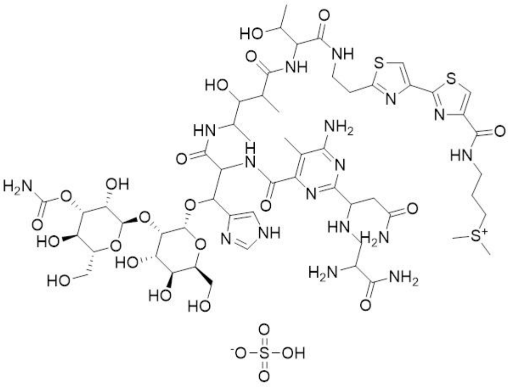

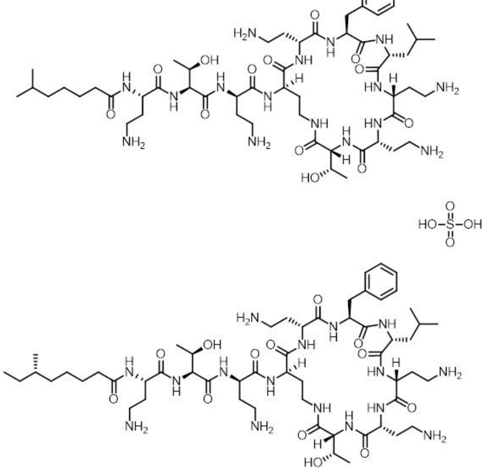

Bleomycin (Sulfate)

Polymyxin B (Sulfate)

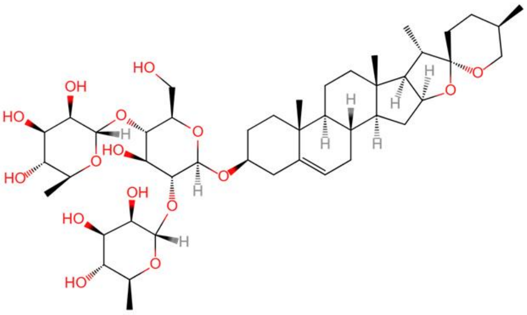

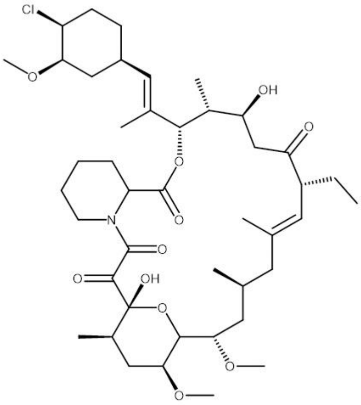

Dioscin

Pimecrolimus

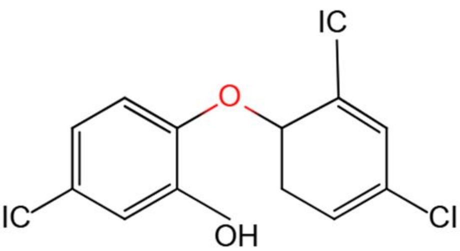

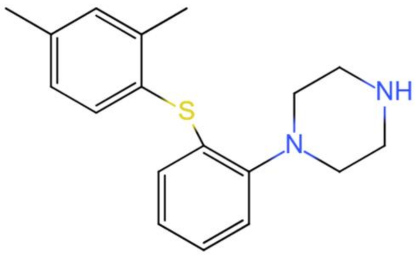

Triclosan

Vortioxetine

Figure S4. The chemical structures of the eight lead compounds.

A

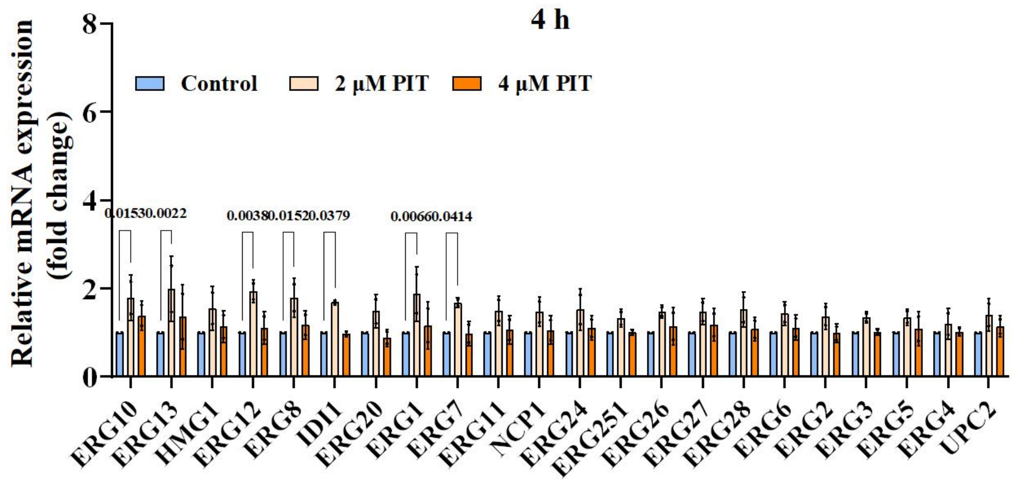

B

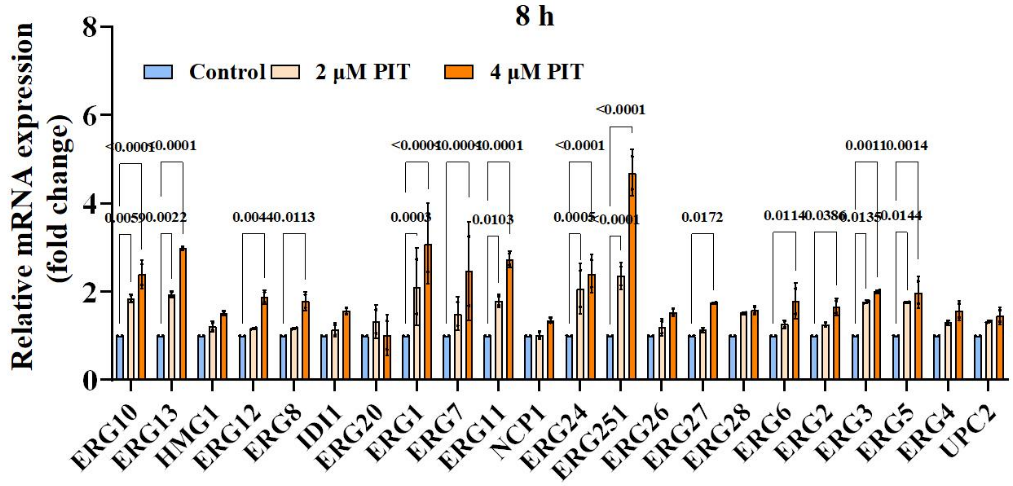

C

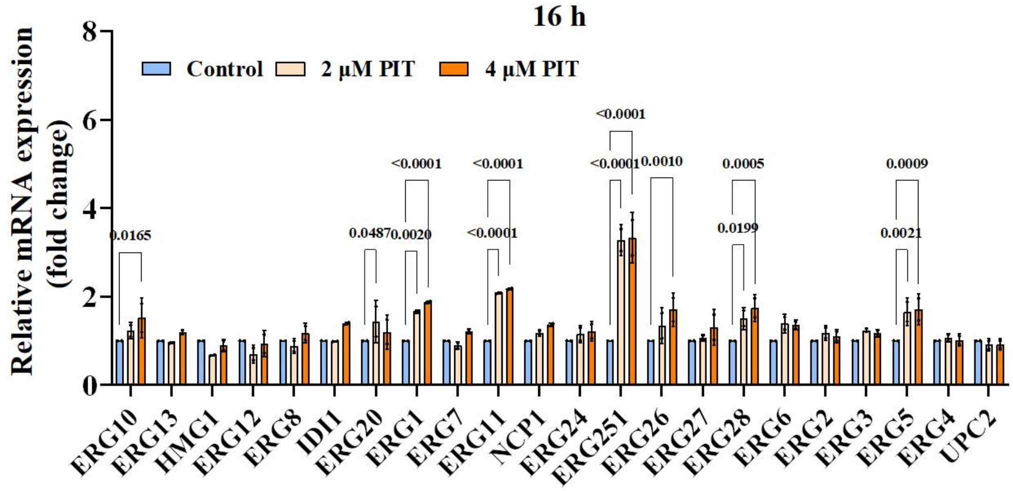

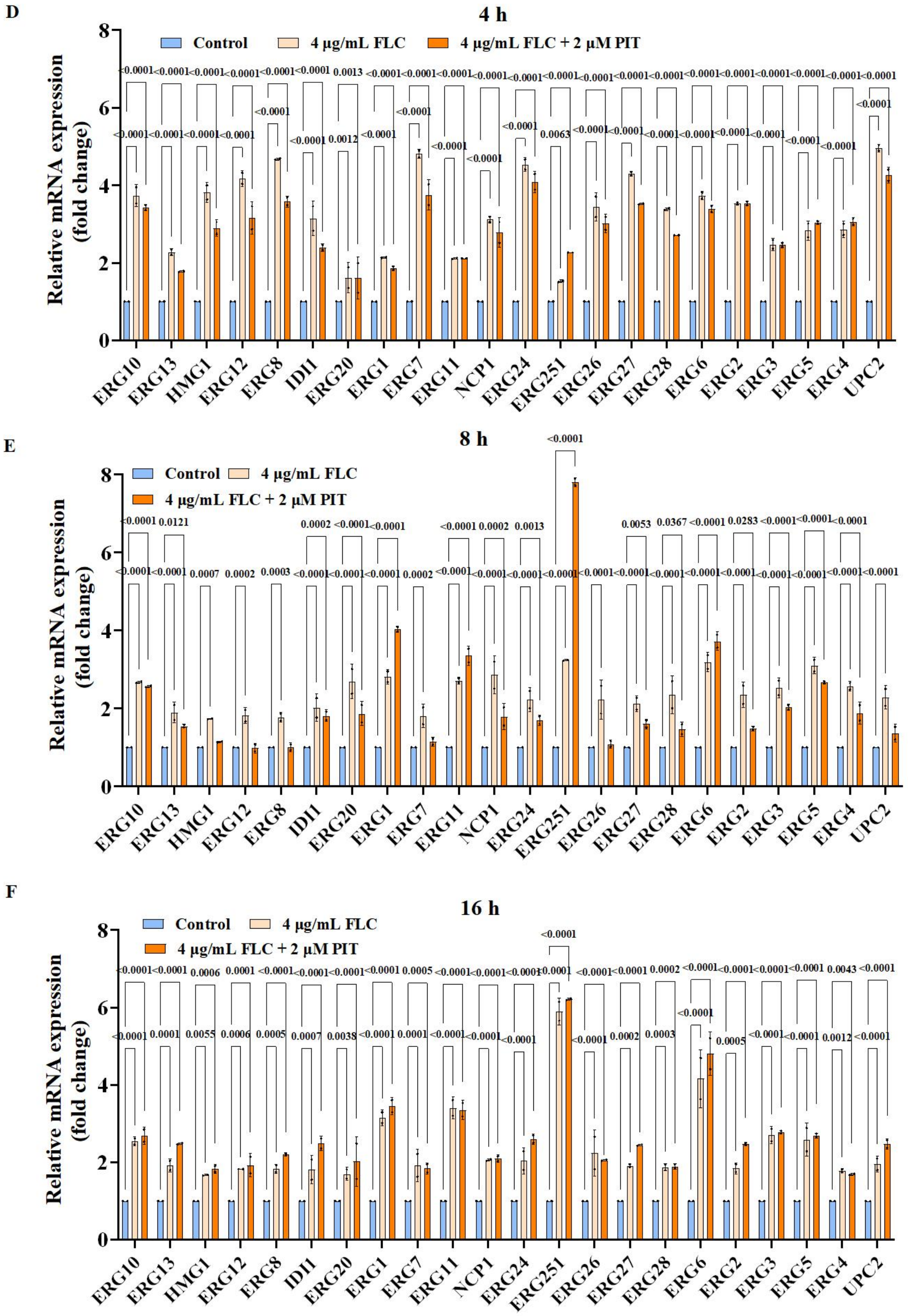

Figure S5. The relative expressions of the ergosterol biosynthesis pathway genes. The wild-type strains were incubated at 30 °C for 4 h **(A)**, 8 h **(B)** or 16 h **(C)** in the presence of PIT (0, 2, 4 μM). The wild-type strains were incubated at 30 °C for 4 h **(D)**, 8 h **(E)** or 16 h **(F)** in YPD medium, YPD medium plus 4 μg/mL FLC, and YPD medium plus

4 µg/mL FLC and 2 µM PIT. The mRNA levels of the *ACT1* gene were used as the control. Two-tailed unpaired *t*-tests and two-way ANOVA were used.

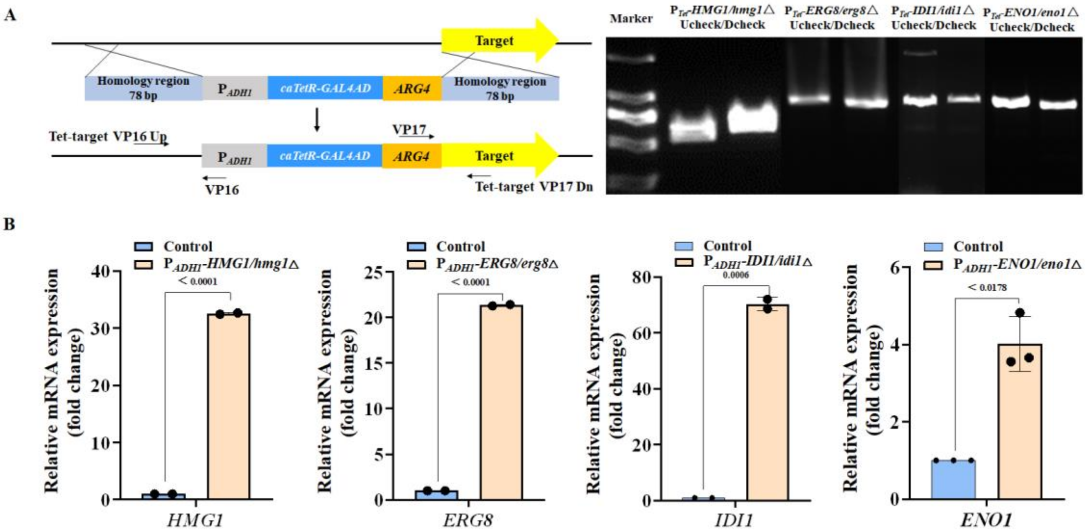

Figure S6. Strains of contained tetracycline-responsive elements were constructed. **(A)** The schematic diagram of tetracycline-responsive elements construction and PCR verification results of P_{Tet}-*HMG1/hmg1*Δ, P_{Tet}-*ERG8/erg8*Δ, P_{Tet}-*IDI1/idi1*Δ. **(B)** qRT-PCR confirmed the ectopic overexpression of the *HMG1*, *ERG8*, *IDI1* genes in P_{ADH1}-*HMG1/hmg1*Δ, P_{ADH1}-*ERG8/erg8*Δ, P_{ADH1}-*IDI1/idi1*Δ mutants. The mRNA levels of the *ACT1* gene were used as the control. Two-tailed unpaired *t*-tests were used.

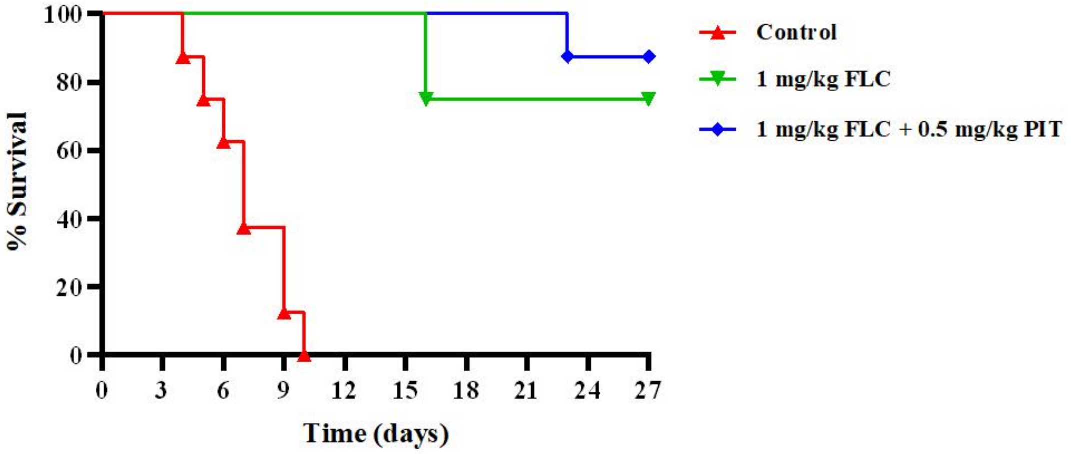

Figure S7. Animal survival analysis of *C. albicans* SN152. C57BL/6 mice were intravenously infected with *C. albicans* SN152. FLC was administered at 1 mg/kg and PIT at 0.5 mg/kg intraperitoneally once daily. The treatment lasted 3 days and monitored the health and survival of treated (green and blue) and untreated (red) mice for 27 days. Significance was determined using the Log-rank (Mantel-Cox) test.

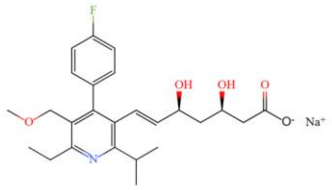

Cerivastatin (sodium)
0.7812 μM

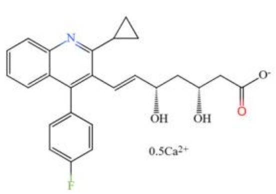

PIT
1.5625 μM

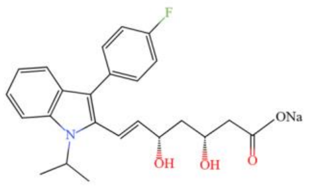

Fluvastatin (sodium)
6.25 μM

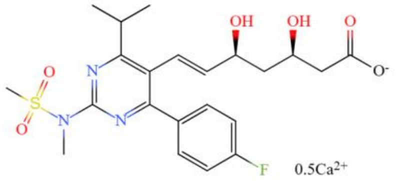

Rosuvastatin (Calcium)
6.25 μM

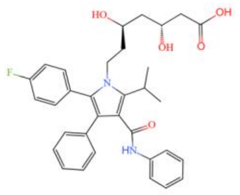

Atorvastatin
> 100 μM

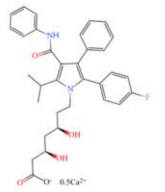

Atorvastatin (hemicalcium salt)
> 100 μM

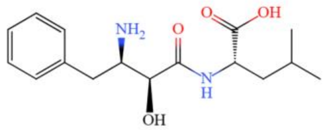

Bestatin
> 100 μM

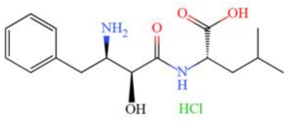

Bestatin (hydrochloride)
> 100 μM

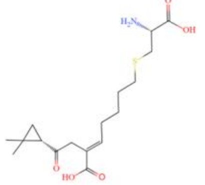

Cilastatin
> 100 μM

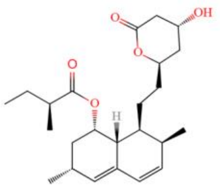

Lovastatin
> 100 μM

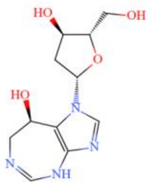

Pentostatin
> 100 μM

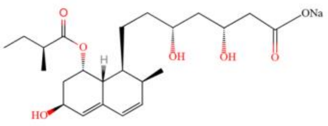

Pravastatin (sodium)
> 100 μM

Figure S8. Structures and synergistic lethal concentrations of statins with FLC.
